# Factors Impacting Invader-Mediated Recognition of Double-Stranded DNA

**DOI:** 10.3390/molecules28010127

**Published:** 2022-12-23

**Authors:** Caroline P. Shepard, Raymond G. Emehiser, Saswata Karmakar, Patrick J. Hrdlicka

**Affiliations:** Department of Chemistry, University of Idaho, Moscow, ID 83844-2343, USA

**Keywords:** oligonucleotides, DNA recognition, chromosomes, DNA, pyrene, fluorescence, FISH, karyotyping, SNP, strand invasion

## Abstract

The development of chemically modified oligonucleotides enabling robust, sequence-unrestricted recognition of complementary chromosomal DNA regions has been an aspirational goal for scientists for many decades. While several groove-binding or strand-invading probes have been developed towards this end, most enable recognition of DNA only under limited conditions (e.g., homopurine or short mixed-sequence targets, low ionic strength, fully modified probe strands). Invader probes, i.e., DNA duplexes modified with +1 interstrand zippers of intercalator-functionalized nucleotides, are predisposed to recognize DNA targets due to their labile nature and high affinity towards complementary DNA. Here, we set out to gain further insight into the design parameters that impact the thermal denaturation properties and binding affinities of Invader probes. Towards this end, ten Invader probes were designed, and their biophysical properties and binding to model DNA hairpins and chromosomal DNA targets were studied. A Spearman’s rank-order correlation analysis of various parameters was then performed. Densely modified Invader probes were found to result in efficient recognition of chromosomal DNA targets with excellent binding specificity in the context of denaturing or non-denaturing fluorescence in situ hybridization (FISH) experiments. The insight gained from the initial phase of this study informed subsequent probe optimization, which yielded constructs displaying improved recognition of chromosomal DNA targets. The findings from this study will facilitate the design of efficient Invader probes for applications in the life sciences.

## 1. Introduction

Over the past several decades, numerous chemically modified oligonucleotides and nucleic acid mimics have been designed to target specific sequences of double-stranded DNA (dsDNA) and identify, regulate, and manipulate genes. For example, traditional peptide nucleic acids (PNAs) [1,2] and triplex-forming oligonucleotides (TFOs) [3,4] bind in the major groove of double-stranded DNA (dsDNA), forming Hoogsteen base pairs (bps), which require the presence of extended purine tracts. Pyrrole-imidazole polyamides, on the other hand, have been designed to target complementary sites through binding via the minor groove. However, it has proven challenging to design polyamides that target sufficiently long sequences, as shape complementarity in the minor groove gradually vanishes with increasing probe length [5,6].

Strand-invading approaches—i.e., chemically modified oligonucleotides and nucleic acid mimics capable of unzipping Watson-Crick base pairs of dsDNA targets and forming new, more stable Watson-Crick base pairs between probe strands and the complementary DNA (cDNA) regions—have been explored to overcome the limitations of groove-binding approaches. A key advantage of strand-invading strategies is the prospect of the sequence-unrestricted recognition of dsDNA. Progress towards this end has been realized with various modified single-stranded PNAs [7,8,9,10,11,12], double-stranded probes such as pseudo-complementary (pc) PNAs [13,14,15], and related approaches [16,17,18,19,20,21,22,23].

**Figure 1:**
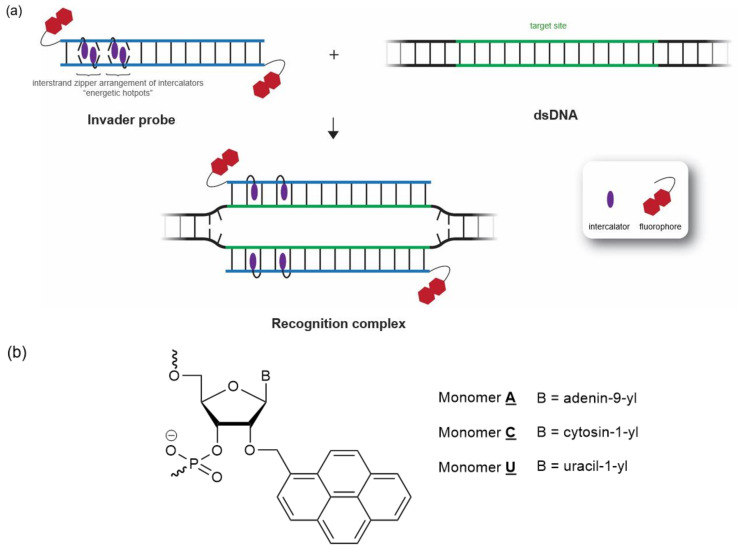
(**a**) Illustration of Invader-mediated recognition of dsDNA via a double-duplex invasion process. (**b**) Structures of Invader monomers used herein.

We focused on the development of dsDNA-targeting Invader probes [24], i.e., short DNA duplexes featuring one or more +1 interstrand zipper arrangements [25] of intercalator-functionalized nucleotides such as 2′-*O*-(pyren-1-yl)methyl-RNA (Figure 1). This monomer arrangement—coined an *energetic hotspot* for brevity—forces pairs of intercalators between the π-stacks of neighboring base pairs in the double-stranded probe, resulting in a violation of the neighbor exclusion principle [26]. The principle asserts that local intercalator densities exceeding one intercalator per two base pairs are unfavorable in DNA duplexes due to limitations in local helix expandability (each intercalation event expands the duplex by ~3.4 Å), and because stabilizing stacking interactions between neighboring base pairs and the first intercalating moiety are perturbed [27,28,29]. Accordingly, double-stranded Invader probes, featuring two intercalators between the two base pairs of the hotspot region, are partially unwound and labile (Figure 1) [30,31]. The two Invader probe strands, in turn, display high affinity towards cDNA, as duplex formation results in strongly stabilizing stacking interactions between the intercalator and flanking base pairs (the neighbor exclusion principle is no longer violated, as the local intercalator density is one intercalator per two base pairs or less). The difference in stability between the probe-target duplexes, vis-à-vis the double-stranded Invader probe and the dsDNA target region, generates the driving force for dsDNA recognition via double-duplex strand invasion (Figure 1) [24].

The sequence-unrestricted recognition of dsDNA targets using Invader probes has been demonstrated, enabling the detection of (i) DNA fragments from specific food pathogens using sandwich assays [32], (ii) telomeric DNA of individual chromosomes in metaphasic spreads [33], and (iii) sex chromosome-specific targets in interphase and metaphase nuclei under non-denaturing conditions [23,24].

In addition to our efforts aimed at optimizing Invader probes through the refinement of the monomer and probe architectures [34,35,36], early foundational studies provided some insight into the design parameters that impact the dsDNA-recognition efficiency of Invader probes [24,37]. For example, the use of intercalator-functionalized pyrimidine monomers (and avoidance of the corresponding guanine monomers) was found to be preferable for the construction of energetic hotspots. This is because the resulting probe-target duplexes are particularly stabilized when the intercalator-modified monomers are flanked by 3′-purines, thus, increasing the thermodynamic driving force for dsDNA-recognition [37].

In the present study, we set out to gain further insight into the design parameters that impact denaturation properties, driving forces for target recognition, and recognition of chromosomal DNA targets. Towards this end, a library of Invader probes was constructed; their denaturation, thermodynamic and dsDNA-targeting properties were studied; and a Spearman’s rank-order correlation analysis of different parameter pairs was performed. The insights gained from the initial phase of this study informed the subsequent optimization of probes, which displayed improved recognition of chromosomal DNA targets.

## 2. Results and Discussion

### 2.1. Invader Probe Design

Initially, ten 5′-Cy3-labeled oligodeoxyribonucleotide (ON)-based Invader probes (**INV1**-**INV10**, Table 1), varying in length (14–16 base pairs) and GC-content (GC%) (30–70%), were designed to target complementary sequences within the DYZ-1 satellite gene (~6 × 10^4^ tandem repeats of a ~1175 bp region) located on the bovine (Bos taurus) Y chromosome [38] (NCBI code: M26067, Figure S1). The probes were designed to have modification densities (mod%) of ~20–30%, as earlier studies suggested this level of modification to strike a favorable balance between binding affinity and binding specificity [24,33]. Individual probe strands were obtained using established machine-assisted solid-phase ON synthesis protocols [37].

### 2.2. Thermal Denaturation Properties of Invader Probes

Thermal denaturation temperatures (*T*_m_s) were determined for the double-stranded Invader probes and the corresponding duplexes between individual probe strands and complementary DNA (Table 1). With the exception of **INV2**, **INV5**, and **INV9**, the Invader probes display substantially similar *T*_m_s as the corresponding unmodified DNA duplexes (see Δ*T*_m_s for probe duplexes, Table 1). Conversely, duplexes between individual Invader probe strands and cDNA display *T*_m_s that, on average, are ~13 °C higher than the corresponding unmodified DNA duplexes with Δ*T*_m_s ranging between +3.5 and +22.0 °C (see Δ*T*_m_ values for 5′-ON:cDNA and 3′-ON:cDNA, Table 1). The observed differences in *T*_m_ values are in agreement with prior results [24] and reflect that the neighbor exclusion principle is violated in the double-stranded Invader probes (high local intercalator density) but not in duplexes between individual Invader strands and cDNA (lower local intercalator density).

Our Spearman’s rank-order correlation analysis of select parameter pairs (full dataset in Supplementary Materials) indicates that there is a lack of significant correlation between the Δ*T*_m_ values of the Invader probes and any of the following parameters: length, GC-content, modification density, number of modifications (#mod), or longest unmodified stretch (stretch), at least within the pre-selected design restrictions of the test set (*p* >> 0.05, entries 1–5, Table 2). In contrast, a significant positive correlation between the Δ*T*_m_ values of the probe–cDNA duplexes and modification density was observed (*p* < 0.05, *r*_s_ >> 0, entries 6 and 7, Table 2). This, along with a significant negative correlation with the longest unmodified stretch metric (*p* < 0.05, *r*_s_ << 0, entries 8 and 9, Table 2), suggests that densely modified Invader probes with short unmodified stretches yield probe-target duplexes displaying the most prominent increases in *T*_m_ values relative to the corresponding unmodified DNA duplexes. Accordingly, average Δ*T*_m_s of ~17 °C and ~10 °C were observed for probe:cDNA duplexes entailing probe strands with modification densities of >20% and ≤20%, respectively, Table 1). Moreover, negative correlations approaching significance were observed between the Δ*T*_m_ values of probe-target duplexes and the GC-content or *T*_m_ of the corresponding unmodified DNA duplexes (entries 10–13, Table 2). This suggests that the stabilizing impact of the 2′-*O*-(pyren-1-yl)methyl-RNA monomers in probe:cDNA duplexes is more pronounced when Invader probes are designed to target lower melting AT-rich regions. Accordingly, probe–cDNA duplexes with lower GC-content display greater relative increases (Δ*T*_m_ ~16 °C and ~9.5 °C for duplexes with GC% of <50% and ≥50%, respectively, Table 1).

### 2.3. Driving Force for Recognition of dsDNA Targets

The driving force for the Invader-mediated recognition of isosequential dsDNA targets—i.e., complementary DNA duplexes of identical length and sequence—can be assessed using *T*_m_- or Δ*G*-based terms. Concerning the former, we define the thermal advantage as TA = 5′-ON:cDNA Δ*T*_m_ + 3′-ON:cDNA Δ*T*_m_—probe duplex Δ*T*_m_. Thus, prominently positive TA values are expected for double-stranded probes that are activated for the recognition of isosequential dsDNA targets. Indeed, eight of the ten Invader probes display TA values greater than 20 °C, indicating that these probes are activated for the recognition of complementary dsDNA regions (Table 1).

The Spearman rank-order correlation analysis of the dataset indicates that there are correlations approaching significance between *TA* values and the metrics used to calculate the term (entries 14—16, Table 2). Moreover, a strongly negative correlation was observed between *TA* and Invader *T*_m_ values (entry 17, Table 2), indicating that low-melting Invader probes exhibit the most pronounced driving forces for the recognition of dsDNA. However, there is no correlation between *TA* values and GC-content (entry 18, Table 2). Importantly, a significant positive correlation between *TA* values and modification density or number of modifications was observed (entries 19 and 20, Table 2). Accordingly, the quadruply and most densely modified Invader probes display the most prominent *TA* values (*TA*s between 38.0 and 44.5 °C for **INV2**, **INV7** and **INV10**, Table 1).

Alternatively, the available free energy for the recognition of an isosequential dsDNA target at 310 K can be determined as Δ*G*${}_{rec}^{310}$ = Δ*G*^310^ (5′-ON:cDNA) + Δ*G*^310^ (3′-ON:cDNA)—Δ*G*^310^ (probe duplex)—Δ*G*^310^ (dsDNA) (Table 1 and Table S2). Thermodynamic parameters were derived from thermal denaturation curves via the baseline-fitting method (Tables S2–S4) [39]. Prominently negative values indicate a probe with a strong thermodynamic driving force for the recognition of isosequential dsDNA targets. In agreement with the *TA*-based conclusions, Invader probes are prominently activated for dsDNA-recognition (Δ*G*${}_{rec}^{310}$ between −7 and −56 kJ/mol, Table 1). This is due to the labile nature of the Invader probes (ΔΔ*G*^310^ values, calculated relative to the corresponding unmodified dsDNA target, range between −1 kJ/mol and +25 kJ/mol; averaging +12 kJ/mol, Table S2) and the prominent stability of the probe-target duplexes (ΔΔ*G*^310^ values range between +19 kJ/mol and −33 kJ/mol, averaging −7 kJ/mol, Table S2). The driving force for dsDNA-recognition is generally due to favorable changes in enthalpy (Δ*H*_rec_ << 0 kJ/mol, Table S3) [40]. This reflects that the formation of probe duplexes is considerably less enthalpically favorable than the corresponding probe:cDNA duplexes (ΔΔ*H* values range between +174 kJ/mol and +372 kJ/mol, Table S3), which, again, is due to the energetic hotspots and the ensuing violation of the neighbor exclusion principle.

The Spearman rank-order correlation analysis of the dataset confirmed the expected negative correlation between Δ*G*${}_{rec}^{310}$ and *TA* values (entry 21, Table 2), i.e., negative Δ*G*${}_{rec}^{310}$ values correlate with positive *TA* values. Negative correlations approaching significance between Δ*G*${}_{rec}^{310}$ values and modification density or number of modifications were also observed (entries 22 and 23, Table 2). Hence, both the Δ*G*${}_{rec}^{310}$ and *TA* parameters indicate that the thermodynamic gradient for dsDNA recognition is maximized when densely modified Invader probes are used.

### 2.4. Recognition of Mixed-Sequence Model DNA Hairpin Targets

**Figure 2:**
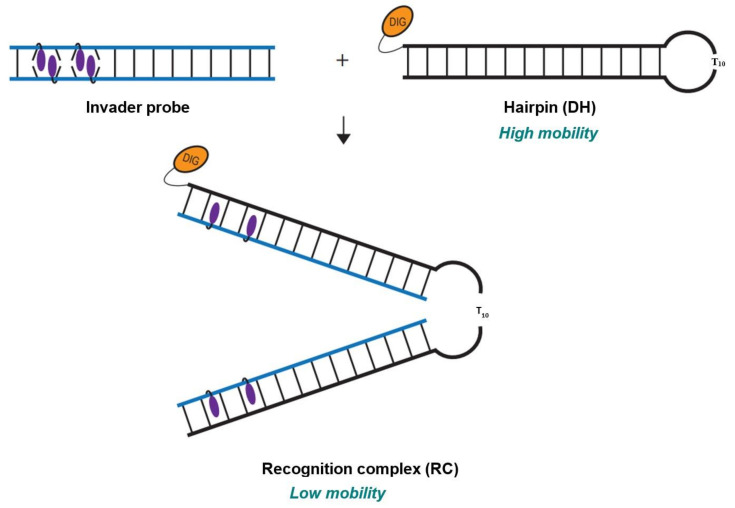
Illustration of EMSA assay used to evaluate dsDNA-recognition of Invader probes.

The dsDNA-recognition characteristics of the initial set of Invader probes were first evaluated using an electrophoretic mobility shift assay (EMSA), in which the probes were incubated with 3′-digoxigenin (DIG)-labeled DNA hairpin (DH) model targets (Figure 2). Each DNA hairpin (**DH1**-**DH10**) comprises a double-stranded stem that is complementary to the corresponding Invader probe, and in which one end is linked by a decameric thymidine (T_10_) loop. The resulting hairpins are high-melting (*T*_m_s for **DH1**-**DH10** between 62 and 82 °C, Table S6). This and the unimolecular nature of the DNA hairpins ensures that both target strands are present in equimolar amounts and unlikely to fray. Invader-mediated recognition of the double-stranded stem region is expected to result in the formation of a ternary recognition complex (RC) that manifests itself as a slower-moving band relative to the DNA hairpin when mixtures are resolved by non-denaturing polyacrylamide gel electrophoresis (nd-PAGE).

**Figure 3:**
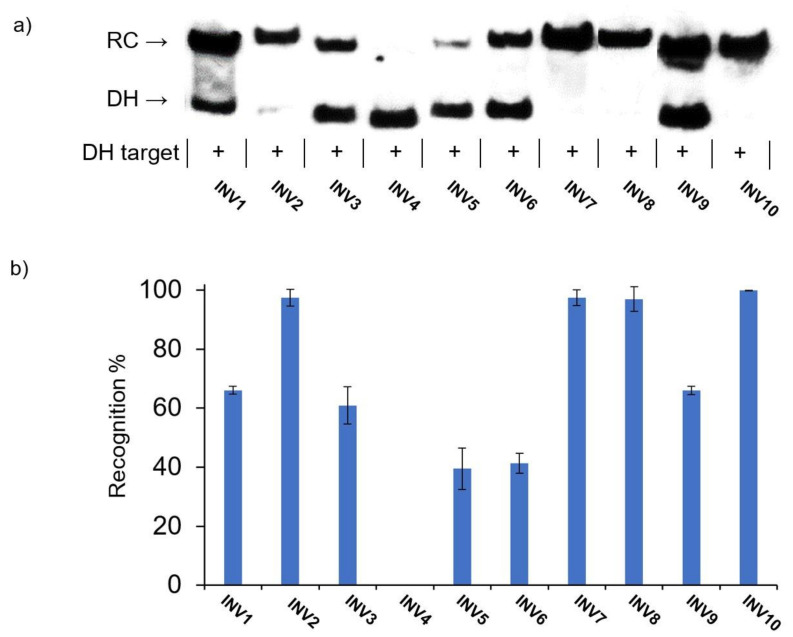
(**a**) Representative electrophoretograms from recognition experiments in which a 100-fold molar excess of Invader probes **INV1**-**INV10** was incubated with their respective DNA hairpin targets **DH1**-**DH10**. (**b**) Histograms depict averaged results from at least three recognition experiments with error bars denoting standard deviation. RC = recognition complex. DH = DNA hairpin. DIG-labeled DNA hairpins **DH1**-**DH10** (34.4 nM, sequences shown in Table S6) were incubated with the corresponding Invader probe in HEPES buffer (50 mM of HEPES, 100 mM of NaCl, 5 mM of MgCl_2_, pH 7.2, 10% sucrose, 1.44 mM of spermine tetrahyrdochloride) at 37 °C for 15 h. Incubation mixtures were resolved on 12% non-denaturing TBE-PAGE slabs (~70 V, ~4 °C, ~1.5 h).

In the initial screen, a 100-fold molar excess of each Invader probe was incubated with the corresponding DNA hairpin target for 15 h at 37 °C in a HEPES buffer containing 100 mM of NaCl and 5 mM of MgCl_2_ (Figure 3) [41]. Essentially complete dsDNA-recognition was observed for four of the ten probes (**INV2**, **INV7**, **INV8**, and **INV10**), while five probes resulted in moderate recognition (40–70%, **INV1**, **INV3**, **INV5**, **INV6**, and **INV9**) (Figure 3 and Table 3). No dsDNA recognition was observed for **INV4**; this was a surprising result considering that this probe has been used to detect chromosomal DNA targets under non-denaturing FISH conditions [24].

**Figure 4:**
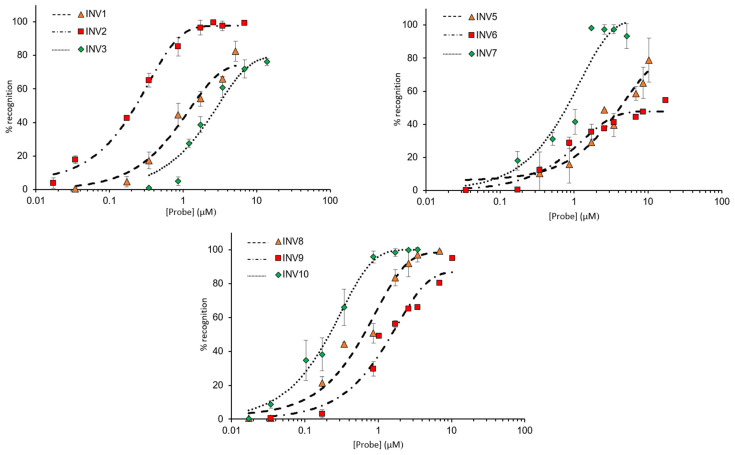
Dose–response curves for recognition of DNA hairpins using **INV1-INV3** (upper left panel), **INV5-INV7** (upper right panel), and **INV8-INV10** (lower panel). Probes were incubated with their respective DNA hairpin targets for 15 h at 37 °C. Experimental conditions are as described in Figure 3, except for variable probe concentrations. Bars denote standard deviations. For the corresponding electrophoretograms, see Figures S11–S13.

Next, dose–response relationships were established to determine C_50_ values, i.e., the probe concentrations resulting in 50% recognition of a corresponding DNA hairpin target (Figure 4). **INV2** and **INV10** displayed the most efficient recognition (C_50_ ~ 0.2 µM, Table 3), followed by **INV7** and **INV8** (C_50_ = 0.6–0.7 µM, Table 3). Moderately efficient recognition was observed for **INV1**, **INV3**, **INV5**, and **INV9** (C_50_ = 1.3–4.1 µM, Table 3), whilst **INV6** only displayed marginal recognition of **DH6** (C_50_ ≥ 10 µM, Table 3).

The Spearman’s rank-order correlation analysis of the dataset indicates the presence of significant correlations between the observed C_50_ values and the modification density, number of modifications, or longest unmodified stretch of the Invader probes (entries 1–3, Table 4). Accordingly, the quadruply and most densely modified **INV2**, **INV7**, and **INV10** probes display the lowest C_50_ values, while all but one of the less densely modified probes (mod% < 21.5%) display moderate or no recognition of DNA hairpin targets (Table 3).

The level of recognition observed for **INV8** is surprising given that it is only ~20% modified (Table 3). However, it should be noted that **INV8** displays favorable *TA* and Δ*G*${}_{rec}^{310}$ values (*TA* = 26.5 °C and Δ*G*${}_{rec}^{310}$ = −52 kJ/mol, Table 1). This is relevant since correlations approaching significance were also observed between C_50_ values and *TA* or Δ*G*${}_{rec}^{310}$ values (entries 4 and 5, Table 4).^r^ Further along these lines, correlations approaching significance were observed between C_50_ values and measures of probe:cDNA duplex stability (entries 6–9, Table 4), indicating that the formation of stable probe-target duplexes is an important driver of DNA hairpin recognition. The relatively high levels of hairpin recognition observed with **INV8** may, therefore, be linked to the high stability of the corresponding probe-target duplexes (Δ*T*_m_ average of +12.5 °C, ΔΔ*G*^310^ averaging −12.5 kJ/mol, Table 1 and Table S2, respectively).

Somewhat surprisingly, no correlation was observed between the C_50_ values and the *T*_m_, Δ*T*_m_, Δ*G*^310^, ΔΔ*G*^310^ values, or the GC-content of the Invader probes (entries 10–14, Table 4). This indicates that the absolute or relative stability of Invader probes—at least within the design constraints of the test set—does not impact hairpin recognition.

**Figure 5:**
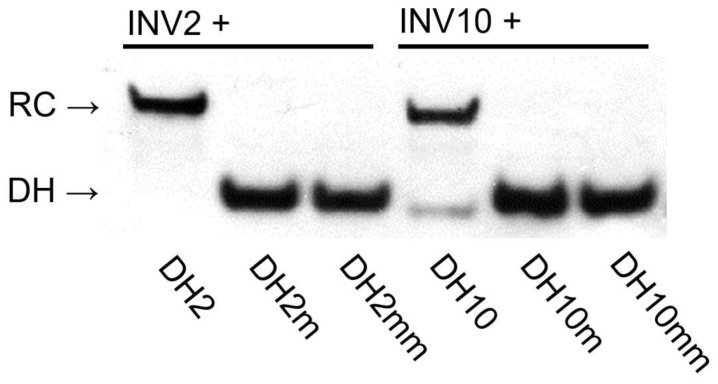
Binding specificity of Invader probes. A 100-fold molar probe excess was incubated with corresponding DNA hairpins featuring stems of identical sequence or differing in sequence at one (“m”) or two positions (“mm”) relative to the probes (37 °C, 15 h). For sequences of DNA hairpins, see Table S6. Conditions are as described in Figure 3. Data previously shown in [23]—reproduced with permission from the Royal Society of Chemistry.

The binding specificities of high-affinity Invader probes were evaluated by incubating a 100-fold molar excess of **INV2** and **INV10** with DNA hairpins featuring stems that differ in sequence at one or two positions relative to the probes (sequences shown in Table S6). Both probes fully discriminated these DNA hairpins, while resulting in complete recognition of the complementary targets (Figure 5). Remarkably, this demonstrates that high-affinity Invader probes can distinguish targets with ~94% sequence homology (i.e., fifteen of the sixteen bps are identical between **DH2** and **DH2m**). This finding hints at interesting single nucleotide polymorphism (SNP) applications for Invader probes.

### 2.5. Targeting Chromosomal DNA—Fluorescence In Situ Hybridization (FISH) Assays

Next, the ten Cy3-labeled Invader probes were evaluated for their ability to recognize corresponding DNA target regions within the *DYZ*-1 gene of the bovine Y chromosome in the context of FISH assays. Thus, INV1–INV10 were incubated with fixed interphase nuclei from a male bovine kidney cell line under denaturing (d) or non-denaturing (nd) FISH conditions. The d-FISH assay was expected to yield information about the maximal recognition capacity of each probe, since access to the chromosomal DNA target regions is facilitated by high incubation temperatures. The nd-FISH experiments, on the other hand, were expected to reveal if a probe can recognize the corresponding target at more physiologically relevant conditions. Successful target recognition was expected to manifest itself in the form of a single, punctate fluorescent signal.

**Figure 6:**
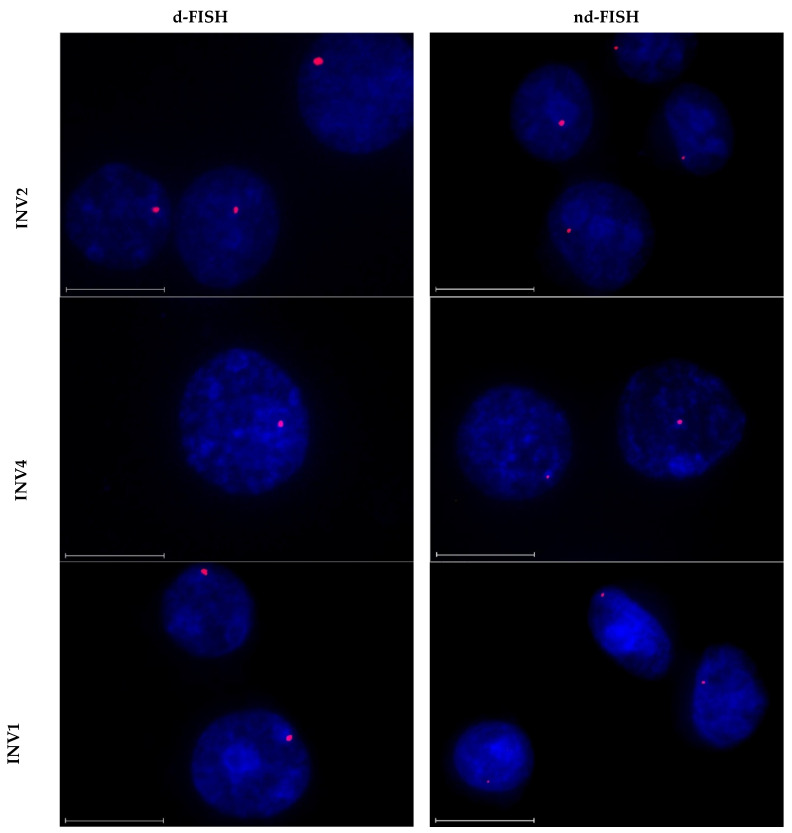
Representative images from FISH experiments in which *DYZ1*-targeting Invader probes **INV2**, **INV4**, and **INV10** (upper, middle and lower panels, respectively) were incubated with isolated nuclei from a bovine kidney cell line under denaturing (5 min, 80 °C, left) or non-denaturing (3 h, 37.5 °C, right) conditions. Fixed isolated nuclei were incubated with probes in a Tris buffer (20 mM of Tris-Cl, 100 mM of KCl, pH 8.0) and counterstained with DAPI. The images were obtained by overlaying Cy3 (red) and DAPI (blue) filter settings and adjusting the exposure. Nuclei are viewed at 60× magnification using a Nikon Eclipse T*i*-S inverted microscope. The scale bar represents 16 µm. For corresponding images for other Invader probes, see Figures S14–S17.

The two high-affinity probes, **INV2** and **INV10**, were found to recognize the DNA targets with excellent efficiency in d-FISH assays (i.e., ~90% of the analyzed nuclei displayed a single, intense, punctate signal against a low level of background; Figure 6 left column and Table 5). As previously reported [24], excellent target recognition was also observed with **INV4**. This was surprising considering the low driving force for dsDNA-recognition (*TA* = 1.5 °C and Δ*G*${}_{rec}^{310}$ = −7 kJ/mol, Table 1) and the lack of DNA hairpin recognition (Figure 3). While the reasons for the different performance in the DNA hairpin and d-FISH experiments observed with **INV4** are not fully understood, it should be noted that the experimental conditions (e.g., buffers, probe concentrations) are quite different, which may impact probe binding. Five probes (**INV3** and **INV6**-**INV9**) displayed single punctate signals in 40–60% of the analyzed nuclei under d-FISH conditions (Table 5 and Figures S15–S17 left column). Two probes failed to yield acceptable signal profiles, i.e., **INV1,** resulting in the formation of multiple signal blotches indicative of non-specific binding, and **INV5,** which did not produce signals of any kind (Table 5 and Figures S14 and S15, respectively).

The probes largely retained their signaling capacities under nd-FISH conditions. Thus, **INV2**, **INV4**, and **INV10** yielded single, intense, punctate signals against a low background in 85%−90% of the analyzed nuclei (Figure 6 right column and Table 5). Moderately intense signals were observed for four of the probes in 20–30% of the nuclei (i.e., **INV3** and **INV6**-**INV8**, Figures S15 and S16, Table 5), while three of the probes (i.e., **INV1**, **INV5**, and **INV9**) did not produce discernable signals (Figures S14, S15 and S17 and Table 5). The diverging results observed for **INV9** under d-FISH vis-à-vis nd-FISH conditions indicate that this target region is inaccessible under non-denaturing conditions. 

Hence, most of the studied Invader probes resulted in adequate-to-excellent recognition of chromosomal DNA targets under d-FISH and nd-FISH conditions. The Spearman’s rank-order correlation analysis revealed that the signaling performance in the d-FISH and nd-FISH assays significantly correlates with the modification level of the probes and the observed C_50_ values (entries 15–18, Table 4). Along similar lines, correlations approaching significance were observed between the signaling performance in d-FISH and nd-FISH assays and the number of modifications or longest unmodified stretch (entries 19–22, Table 4). Correlations approaching significance were observed between nd-FISH signaling performance and *TA* and Δ*G*${}_{rec}^{310}$ values, indicating that these metrics have some predictive value for nd-FISH, but not d-FISH, performance (entries 23–26, Table 4). Interestingly, signaling performance did not correlate with the GC-content of the target region (entries 27 and 28, Table 4).

The observed correlation with modification density provides a rationale for the excellent signaling characteristics of **INV2** and **INV10** (25–29% modified) and the moderate-to-poor signaling characteristics of most of the remaining probes. The signaling properties of two probes, i.e., **INV4** and **INV7**, however, are not easily rationalized. Thus, excellent signaling properties were observed for the sparsely modified **INV4** that failed to recognize the corresponding DNA hairpin target (Figure 3) and was far less activated for dsDNA-recognition than **INV2** and **INV10** (compare *TA* and Δ*G*${}_{rec}^{310}$ values, Table 1). A distinguishing feature of **INV4** and its corresponding target region is the presence of two GGG/CCC-tracts, which we speculate may render the target region uniquely accessible due to the formation of non-canonical secondary structures [42]. An alternative explanation for the surprising signaling characteristics of **INV4** is that the corresponding target region is present six times within a single *DYZ−1* repeat (which, in turn, is repeated ~6 × 10^4^ times, Figure S1) [24], whilst the other target regions studied herein are only present once per *DYZ−1* repeat. The greater number of target sites may account for cooperative hybridization effects, resulting in a greater proportion of nuclei that present a signal. The modest signaling properties of **INV7** are perplexing given its high level of modification (25%), prominent activation for dsDNA-recognition (*TA* = 38 °C and Δ*G*${}_{rec}^{310}$ = −46 kJ/mol, Table 1), and efficient hairpin recognition (C_50_ = 0.7 µM, Table 3). We speculate that the corresponding chromosomal DNA target region is only partially accessible to **INV7** under these experimental conditions.

Control nd-FISH experiments, in which fixed nuclei were pre-treated with DNase I, RNase A, or Proteinase K prior to incubation with **INV2** or **INV10**, confirmed that the Invader probes target chromosomal DNA, rather than RNA or proteins. Thus, nuclei that were pre-treated with DNase I did not produce any signals (Figure S18), whereas pre-treatment with RNase A or Proteinase K continued to yield single punctate signals, albeit with lower intensity (Figure S19) [43].

**Figure 7:**
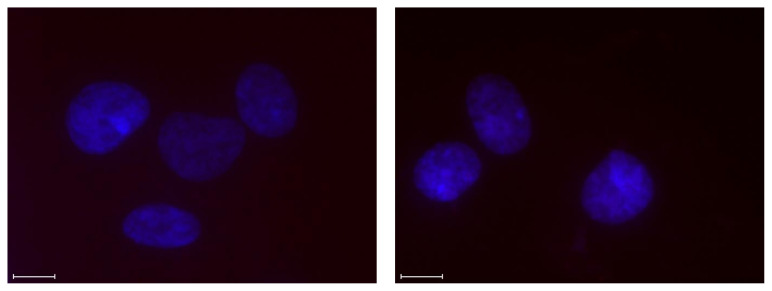
Images from d-FISH experiments in which **INV2** (left panel) and **INV10** (right panel) were incubated with fixed isolated female bovine endothelial nuclei. Note the absence of Cy3 signals. Incubation conditions and the image capture process were as described in Figure 6. Data previously shown in [23]—reproduced with permission from the Royal Society of Chemistry.

Incubation of the Y-chromosome-targeting probes **INV2** and **INV10** with a female bovine endothelial cell line failed to produce signals under denaturing conditions (Figure 7), suggesting that Invader probes bind their chromosomal DNA targets with excellent specificity [43].

### 2.6. Design of Optimized Invader Probes

Having identified modification density as a key parameter for successful dsDNA-recognition, we set out to optimize three Invader probes that displayed poor-to-moderate signaling characteristics under nd-FISH conditions, i.e., **INV6**, **INV8**, and **INV9**. Thus, two or three additional hotspots were introduced to yield probes with modification densities of 27–33% (**OPT6**, **OPT8**, and **OPT9**, Table 1).

### 2.7. Thermal Denaturation and Thermodynamic Properties of Optimized Invader Probes

The more densely modified probes were found to be considerably less stable than the parent probes (*T*_m_s ~20 °C lower and Δ*G*^310^ values ~12 kJ/mol higher on average; compare *T*_m_ and Δ*G*^310^ values for **INV6/8/9** and **OPT6/8/9**, Table 1, Tables S2 and S9, respectively). Moreover, the densely modified probe strands form more stable duplexes with cDNA than the parent counterparts (*T*_m_s ~6 °C higher and Δ*G*^310^ values ~18 kJ/mol lower on average; compare *T*_m_s and Δ*G*^310^ values, Table 1, Tables S2 and S9, respectively). Consequently, the driving forces for the recognition of isosequential dsDNA targets are substantially larger for the three redesigned probes compared to the parent counterparts (*TA* values between 43.0–55.5 °C vs. 13.5–26.5 °C and Δ*G*${}_{rec}^{310}$ values between −93 kJ/mol and −59 kJ/mol vs. between −52 kJ/mol and −19 kJ/mol, Table 1).

### 2.8. Recognition of Model DNA Hairpin Targets by Optimized Invader Probes

**Figure 8:**
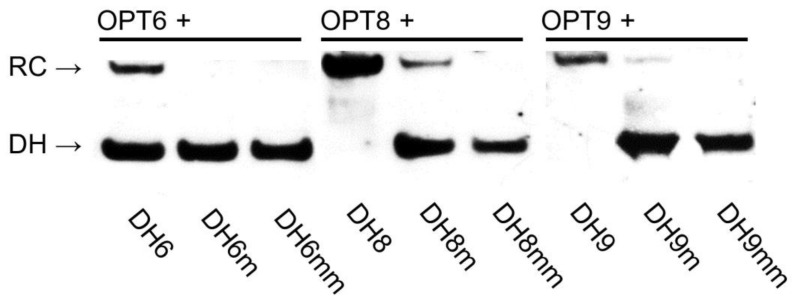
Representative electrophoretograms from recognition experiments in which a 100-fold molar excess of optimized Invader probes **OPT6**, **OPT8**, and **OPT9** was incubated with the corresponding DNA hairpins featuring stems of identical sequence or differing in sequence at one (“m”) or two positions (“mm”) relative to the probes (37 °C, 15 h). For sequences of DNA hairpins, see Table S6.

The dsDNA-recognition characteristics of the three optimized Invader probes were first evaluated using the aforementioned DNA hairpin assay (Figure 2). Thus, the probes were first screened at a 100-fold molar excess (Figure 8 and Figure S22) and then were more fully evaluated in dose–response experiments (Figures S23 and S24). Unlike the corresponding parent probes, **OPT8** and **OPT9** resulted in near-complete recognition of the hairpin targets when incubated at 100-fold molar excess (compare Rec_100x_ values for **OPT8** and **OPT9** vs. **INV8** and **INV9**, Table 3). Surprisingly, **OPT6** resulted in similar levels of recognition of **DH6** as **INV6** (Rec_100x_ ~40%, Table 3). The dose–response experiments verified these findings, as **OPT8** and **OPT9** displayed three- and five-fold reductions in their C_50_ values relative to the parent probes, whilst **OPT6** displayed a C_50_ value > 10 µM (Table 3).

Importantly, complete discrimination of doubly mismatched DNA hairpins and merely trace recognition of the singly mismatched DNA hairpins was observed when the optimized high-affinity **OPT8** and **OPT9** probes were incubated at 100-fold molar excess (Figure 8).

### 2.9. Targeting Chromosomal DNA using Optimized Invader Probes

**Figure 9:**
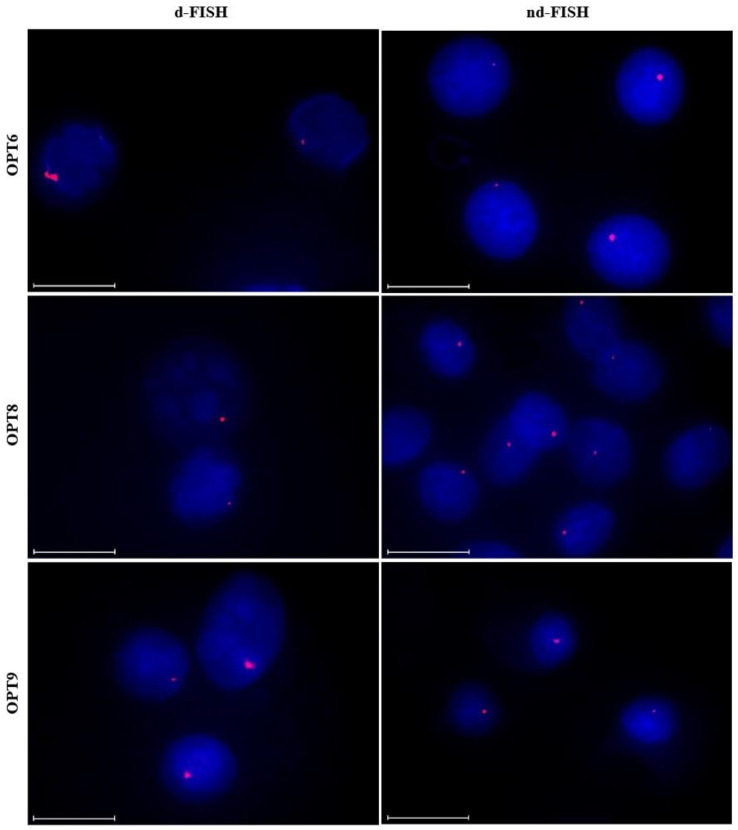
Representative images from FISH experiments in which optimized *DYZ1*-targeting Invader probes **OPT6**, **OPT8**, and **OPT9** (upper, middle, and lower panel, respectively) were incubated with isolated nuclei from a bovine kidney cell line under denaturing (5 min, 80 °C, left) or non-denaturing (3 h, 37.5 °C, right) conditions. Incubation conditions and the image capture process are as described in Figure 6.

The optimized Invader probes were subsequently evaluated for their ability to recognize chromosomal DNA targets using the aforementioned d- and nd-FISH assays. Gratifyingly, improved signaling characteristics, relative to the parent probes, were observed for the optimized probes. Thus, 75%−90% of the nuclei display prominent, single, and punctate signals under d-FISH conditions (Figure 9 left column and Table 5). Along similar lines, ~85%, ~75% and ~25% of the nuclei displayed high-quality signals when **OPT6**, **OPT8** or **OPT9** were used under nd-FISH conditions, respectively, as compared to 0–25% with the parent probes (Figure 9 right column and Table 5). The higher signaling efficiency of **OPT6** vis-à-vis **OPT9** is surprising considering that the latter resulted in far more efficient recognition of the corresponding hairpin target. However, it should be noted that the experimental conditions (e.g., buffers, probe concentrations) are quite different between the two assays, which may impact the results. Nonetheless, the findings demonstrate that increasing the modification density of an Invader probe results in improved signaling characteristics, as per the conclusions of the Spearman’s rank-order analysis. Thus, it is possible to design extensively modified Invader probes that enable sequence-unrestricted and highly specific recognition of chromosomal DNA targets. This important insight will facilitate future biotechnological applications utilizing Invader probes.

## 3. Materials and Methods

### 3.1. Synthesis and Purification of Probe Strands

Individual Invader strands—i.e., oligodeoxyribonucleotides (ONs) modified with 2′-*O*-(pyren-1-yl)methyl-RNA monomers—were synthesized on an Expedite DNA synthesizer (0.2 μmol scale), using columns packed with long-chain alkylamine-controlled pore glass (LCAA-CPG, Glen Research, Sterling, VA, USA) solid support with a pore size of 500 Å. Standard protocols were used for the incorporation of DNA phosphoramidites. The 2′-*O*-(pyren-1-yl)methyl-RNA phosphoramidites were prepared as previously described for U monomer [44] and C/A monomers [37] and incorporated into ONs via extended hand-couplings (15 min, ~45-fold molar excess at a concentration of 0.02 M in anhydrous acetonitrile, using 0.01 M of 4,5-dicanoimidazole as the activator) and oxidation (45 s), resulting in coupling yields of at least 85%. The Cy3-labeling of Invader strands was accomplished by incorporating a commercially available Cy3 phosphoramidite (Glen Research, Sterling, VA, USA) into ONs by hand-coupling (4,5-dicyanoimidazole, 3 min, anhydrous CH_3_CN) (Glen Research, Sterling, VA, USA). Treatment with 32% aq. ammonia (55 °C, 17 h) ensured deprotection and cleavage from solid support. DMT-protected ONs were purified via ion-pair reverse-phase HPLC (Varian, Palo Alto, CA, USA) (Waters, XTerra MS C18 column: 0.05 M of triethyl ammonium acetate and acetonitrile gradient), followed by detritylation (80% acetic acid, 20 min) and precipitation (NaOAc, NaClO_4_, acetone, −18 °C, 16 h). The purity (≥ 85%) and identity of the synthesized ONs was verified using analytical HPLC and MALDI-MS (Tables S1 and S7 and Figures S2–S4 and S16) recorded on a Quadrupole Time-of-Flight (Q-TOF) mass spectrometer (Waters Q-Tof Premier, Milford, MA, USA) using a 3-hydoxypicolinic acid matrix. Common reagents were obtained through VWR International (Radnor, PA) or Fisher Scientific (Hampton, NH).

### 3.2. Thermal Denaturation Experiments

ON concentrations were estimated using the following extinction coefficients (OD_260_/μmol): G (12.01), A (15.20), T (8.40), C (7.05), pyrene (22.4) [45] and Cy3 (4.93) [46]. The thermal denaturation temperatures (*T*_m_s) of the duplexes (1.0 µM final concentration of each strand) were determined on an UV/VIS spectrophotometer (Cary 100, Varian, Palo Alto, CA, USA) equipped with a 12-cell Peltier temperature controller and measured as the maximum of the first derivative of the thermal denaturation curves (*A*_260_ vs. *T*) recorded in medium salt buffer (*T*_m_ buffer: 100 mM of NaCl, 0.2 mM of EDTA, and pH 7.0 adjusted with 10 mM of Na_2_HPO_4_ and 5 mM of Na_2_HPO_4_). Strands were mixed in quartz optical cells with a path length of 1.0 cm and annealed by heating to 85 °C (2 min), followed by cooling to the starting temperature of the experiment. The temperature of the denaturation experiments ranged from at least 15 °C below the *T*_m_ to at least 15 °C above the *T*_m_ (although not above 95 °C). A temperature ramp of 1.0 °C/min was used in all experiments. The reported *T*_m_s are the averages of at least two experiments within ± 1.0 °C.

### 3.3. Electrophoretic Mobility Shift Assays

The non-denaturing (nd)-PAGE assay was performed as previously described [24]. Thus, DNA hairpins (DH) (Integrated DNA Technologies, Coralville, IA, USA) were obtained from commercial sources and used without further purification. Hairpins were 3′-labeled with digoxigenin (DIG) using the 2nd generation DIG Gel Shift Kit (Roche Applied Bioscience, Penzberg, Germany), as recommended by the manufacturer. Briefly, 11-digoxigenin-ddUTP was incorporated at the 3′-end of the hairpin (100 pmol) using a recombinant terminal transferase. The reaction mixture was quenched through the addition of EDTA (0.05 M), diluted to 68.8 nM, and used without further processing. Solutions of Invader probes (concentrations as specified) were incubated with the corresponding DIG-labeled DNA hairpin (final concentration 34.4 nM) in HEPES buffer (50 mM of HEPES, 100 mM of NaCl, 5 mM of MgCl_2_, pH 7.2, 10% sucrose, 1.44 mM of spermine tetrahydrochloride) at 37 °C for the specified time. Following incubation, loading dye (6 ×) was added and the mixtures were loaded onto 12% non-denaturing TBE-PAGE slabs (45 mM of tris-borate, 1 mM of EDTA; acrylamide:bisacrylamide (19:1)). Electrophoresis was performed using constant voltage (~70 V) at ~4 °C for ~1.5 h. The bands were subsequently blotted onto positively charged nylon membranes (~100 V, 30 min, ~4 °C) and cross-linked through exposure to UV light (254 nm, 5 × 15 W bulbs, 5 min). The membranes were then incubated with anti-digoxigenin-alkaline phosphatase F_ab_ fragments, as recommended by the manufacturer, and transferred to a hybridization jacket. They were then incubated with the chemiluminescence substrate (CSPD) for 10 min at 37 °C, and chemiluminescence of the formed product was captured on X-ray films. Digital images of the developed X-ray films were obtained using a BioRad ChemiDoc^TM^ MP Imaging system (BioRad, Hercules, CA, USA), which was also used for densitometric quantification of the bands. The percentage of dsDNA-recognition was calculated as the intensity ratio between the recognition complex band and the unrecognized hairpin. An average of three independent experiments is reported along with standard deviations (±). The presented electrophoretograms are, in some instances, composite images of lanes from different runs. Non-linear regression was used to fit data points from the dose–response experiments. A script written for the “Solver” module in Microsoft Office Excel was used to fit data points from the dose–response experiments to the following equation: y = C + A (1 − e^-kt^) where C, A, and k are fitting constants. The resulting equation was used to calculate C_50_ values by setting y = 50 and solving for t [47].

### 3.4. Spearman Rank-Order Correlation Analysis

A Spearman’s rank-order correlation analysis was performed to identify correlations between parameter pairs and, ultimately, identify parameters that impact the Invader-mediated recognition of dsDNA targets. A wide range of parameters were considered. Spearman rank-order correlation coefficients (*r*_s_) were calculated using the XRealStat function add-on for Microsoft Excel [48]. The ten Invader probes were ranked 1 to 10 for each studied parameter, and these rankings were compared to determine correlations between parameter pairs. For example, the probe with the highest C_50_ and most negative Δ*G*${}_{rec}^{310}$ values would be ranked “1”, while the lowest C_50_ and least negative (or more positive) Δ*G*${}_{rec}^{310}$ values would be ranked 10. Invader probes with identical parameter values received averaged rankings for those parameters. The strength and direction of correlation between two ranked parameters was measured by Spearman’s rank-order correlation coefficient *r*_s_ and deemed statistically significant if the associated *p* values were less than the α value of 0.05.

### 3.5. Cell Culture and Nuclei Preparation

Male bovine kidney cells (MDBK, ATCC: CCL-22, Bethesda, MD, USA) were maintained in DMEM with GlutaMax (Gibco, 10569-010) and 10% fetal bovine serum (Invitrogen, Waltham, MA, USA). Female bovine endothelial cells (CPAE, ATCC: CCL-209) were maintained in Eagle’s Minimum Essential Medium (ATTC, 30-2003) and 20% fetal bovine serum (Invitrogen). The cells were cultured in separate 25 mL or 75 mL flasks at 38.5 °C in a 5% CO_2_ atmosphere for 72–96 h to achieve 70–80% confluency. At this point, colcemid (Gibco KaryoMax, 15210-040) (65 μL per 5 mL of growth media) was added, and the cells were incubated at 37 °C and 5% CO_2_ for an additional 20 min. At this point, the medium was replaced with pre-warmed 0.05% Trypsin-EDTA in DMEM to detach adherent cells (37 °C, up to 8 min). The cell suspension was transferred to a tube and centrifuged (10 min, 1000 rpm). The supernatant was discarded and the dislodged cell pellet was incubated with a hypotonic 75 mM KCl solution (5–8 mL, 20 min), followed by the addition of fixative (10 drops, MeOH:AcOH, 3:1 *v*/*v*) and further incubation with gentle mixing (10 min, room temperature). The suspension was centrifuged (1000 rpm, 10 min), the supernatant discarded, and additional fixative solution (5–8 mL) added to the nuclei suspension. This was followed by gentle mixing and incubation (30 min, room temperature). The centrifugation/resuspension/incubation with fixative solution steps was repeated three additional times. The final pellet—containing somatic nuclei—was resuspended in the fixative solution and stored at −20 °C until use.

### 3.6. Preparation of Slides for FISH Assays

The nuclei suspension was warmed to room temperature and resuspended in fresh fixative solution. Glass microscope slides were dipped in distilled water to create a uniform water layer across the slide. An aliquot of the nuclei suspension (3–5 μL or enough to cover the slide) was dropped onto the slide, while holding the slide at a 45° angle, and allowed to run down the length of the slide. The slides were then allowed to dry at a ~20° angle in an environmental chamber at 28 °C and a relative humidity of 38%.

### 3.7. Fluorescence In Situ Hybridization Experiments and Image Analysis

An aliquot of labeling buffer (~200 μL) consisting of 30 ng of Cy3-labeled Invader probe per 200 μL of PCR buffer (20 mM of Tris, 100 mM of KCl, pH 8.0) placed on each slide. Preliminary assay optimization studies (results not shown) revealed that this “1 × solution” resulted in the best qualitative signal-to-background ratio for the Invader probes under denaturing and non-denaturing conditions. As an exception hereto, **INV4** was used at 0.25× concentration to reduce background fluorescence.

When used in d-FISH assays, slides with labeling buffer were placed on a heating block (5 min, 80 °C) and covered with a lid to prevent evaporation of the labeling buffer. When used in nd-denaturing FISH assays, slides with labeling buffer were placed in a glass culture disk, covered with a lid, and incubated in an oven (3 h, 37.5 °C). Slides for both d-FISH and nd-FISH experiments were subsequently washed (3 min, 37.5 °C) in a chamber with TE Buffer (10 mM of Tris, 1 mM of EDTA, pH 8.0) and allowed to dry at room temperature. Once dried, Gold SlowFade plus DAPI (3 μL, Invitrogen, Waltham, MA, USA) was placed directly on each slide, and a round glass coverslip was mounted for fluorescence imaging. A Nikon Eclipse Ti-S Inverted Microscope (Nikon Instruments, Melville, NY, USA), equipped with a SOLA SMII LED light source system and Cy3 and DAPI filter sets, was used to visualize nuclei at 60× magnification. Images of the fluorophore labeled nuclei were captured using a 14-bit CoolSNAP HQ2 cooled CCD camera and processed with NIS-Elements BR 4.20 software.

Control experiments, in which fixed nuclei from the MDBK cells were pre-treated with DNase, RNase, or proteinase prior to incubation with Invader probes, were carried out as follows. DNase pre-treatment: 3 µL of cloned RNase-free DNase I (Takara N101 JF) was mixed with 50 µL 1 × Reaction Buffer (diluted 10 × Cloned DNase I Buffer II, Takara A301) per the manufacturer’s recommendation. The solution was pipetted onto slides with fixed nuclei in 50 µL amounts. The slides were incubated with the DNase I solution for 20 min at 37.5 °C and then rinsed with TE buffer. RNase pre-treatment: 1 µL of RNase A (5 mg/mL, Fisher reagents BP2539-100) in 100 µL of buffer (10 mM of Tris-HCl, pH 6.5) was placed in 50 µL amounts on slides and incubated for 15 min at 37.5 °C and then rinsed with TE buffer. Proteinase pre-treatment: 1 µL of Proteinase K (6.25 µg/mL, Fisher BioReagents, BP1700-100) was added to 200 µL of buffer (10 mM of Tris-HCl, pH 7.5). The fixed nuclei were incubated with 50 µL of this solution for 10 min at 37.5 °C and then rinsed with TE buffer.

The assessment of signal coverage, i.e., the percentage of nuclei displaying representative signals, was based on an evaluation of >100 nuclei per Invader probe at d-FISH and nd-FISH assay conditions (Table 5).

## 4. Conclusions

Invader probes, i.e., DNA duplexes featuring +1 interstrand zipper arrangements of intercalator-functionalized nucleotides such as 2′-*O*-(pyren-1-yl)methyl-RNA, allow for the robust and highly specific, mixed-sequence recognition of complementary double-stranded DNA target regions. Thus, the successful recognition of a series of model DNA hairpins and chromosomal DNA regions is demonstrated. The modification density is the single-most important design parameter impacting the thermal denaturation and dsDNA-recognition properties of Invader probes. Thus, four of six densely modified Invader probes (modification densities ≥25%) displayed particularly promising signaling characteristics in FISH assays under non-denaturing conditions, i.e., the formation of intense, single, punctate signals against a low fluorescence background in ≥75% of isolated interphase nuclei. The signaling performance is not limited by the GC-content of the target regions, as successful recognition was demonstrated for target regions with GC-contents between 36% and 71%. The modification density also impacts signaling performance in denaturing FISH assays, the efficiency of DNA hairpin recognition, as well as metrics quantifying the driving force for dsDNA-recognition (i.e., *TA* and Δ*G*${}_{rec}^{310}$ values) and the stability of probe-target duplexes (i.e., Δ*T*_m_ or ΔΔ*G*^310^ values for probe-target duplexes). In contrast, the modification density has a limited impact on the stability of the probe (i.e., Δ*T*_m_ or ΔΔ*G*^310^ values for probe duplexes). We speculate that a high modification density results in a perturbed probe that exposes the pyrene moieties, allowing them to contact the target dsDNA and initiate the unwinding process. Identification of the modification density as a key design parameter enabled improvement of three Invader probes with mediocre signaling characteristics in nd-FISH assays into probes displaying improved signaling performance.

Based on the findings from the present and prior studies, we offer the following recommendations for the design of nd-FISH Invader probes:

(i) Invader probes should be densely modified (≥25%) and only feature short unmodified segments. Given the nature of the energetic hotspots (i.e., +1 interstrand zipper arrangements of 2′-*O*-(pyren-1-yl)methyl RNA monomers), an Invader probe can, at most, be 50% modified. Invader probes display exceptional binding specificity, though binding to singly mismatched dsDNA targets may be observed for very densely modified probes. If non-specific binding is observed, the modification density should be reduced. 

(ii) The energetic hotspots of Invader probes should be constructed using 2′-*O*-(pyren-1-yl)methyl RNA pyrimidine monomers, whilst the corresponding guanine monomers are to be avoided; the adenine monomers are acceptable [37]. This maximizes the driving force for dsDNA-recognition as particularly stable probe-target duplexes are formed, since the intercalating pyrene moiety stacks strongly with 3′-flanking purines [37]. Thus, it is recommended that 5′-BC-3′ steps (and B = G in particular) are omitted for the introduction of energetic hotspots. This sets the practical upper limit of the probe’s modification density [49].

These design recommendations, coupled with the straightforward synthesis of the requisite 2′-*O*-(pyren-1-yl)methyl RNA pyrimidine building blocks [37,44], is expected to facilitate the design and use of Invader probes for a broad range of applications in the life sciences.

## Figures and Tables

**Table 1:** Thermal denaturation temperatures (*T*_m_s) of Invader probes and duplexes between individual probe strands and cDNA. Also shown are the length, thermal advantage values (*TA*), available free energy for recognition of isosequential dsDNA targets at 310 K (Δ*G*${}_{rec}^{310}$), modification densities (mod%), and GC-content (GC%) of Invader probes ^a^.

.	.	*T_m_* [Δ*T_m_*] (°C)			.			
Probe (Length)	Sequence	Probe Duplex	5′-ON: cDNA	3′-ON: cDNA	*TA* (°C)	Δ*G* ${}_{rec}^{310}$ (kJ/mol)	mod%	GC%
**INV1** **(15)**	5′-Cy3-T**U**ATCAGCAC**U**G**U**GC-3′	52.0 ^b^	65.5	66.0	+23.5	ND	20.0%	46.7%
	3′-AA**U**AGTCGTGA**C**A**C**G-Cy3-5′	[−4.0]	[+9.5]	[+10.0]				
								
**INV2** **(16)**	5′-Cy3-A**U**AC**U**GGTTTG**U**G**U**TC-3′	34.5 ^b^	66.0	66.0	+44.5	−50	25.0%	37.5%
	3′-TA**U**GA**C**CAAACA**C**A**A**G-Cy3-5′	[−18.5]	[+13.0]	[+13.0]				
								
**INV3** **(15)**	5′-Cy3-T**U**G**U**GCCCTGGC**A**AC-3′	NT	64.0	62.0	ND	ND	20.0%	60.0%
	3′-AA**C**A**C**GGGACCGT**U**G-Cy3-5′		[+5.5]	[+3.5]				
								
**INV4** **(14)**	5′-Cy3-**A**GCCCUGTGCCCTG-3′	61.5	69.5	75.5	+23.0	−7	21.4%	71.4%
	3′-T**C**GGGA**C**ACGGG**A**C-Cy3-5′	[+1.0]	[+9.0]	[+15.0]				
								
**INV5** **(16)**	5′-Cy3-G**A**TTTCAGCCAUGUGC-3′	45.0	63.0	69.5	+30.5	−29	18.8%	50.0%
	3′-CT**A**AAGTCGGTA**C**A**C**G-Cy3-5′	[−12.0]	[+6.0]	[+12.5]				
								
**INV6** **(16)**	5′-Cy3-C**U**G**U**GCAACTGGT**U**TG-3′	63.0	65.5	69.0	+13.5	−22	18.8%	50.0%
	3′-GA**C**A**C**GTTGACCAA**A**C-Cy3-5′	[+5.0]	[+7.5]	[+11.0]				
								
**INV7** **(16)**	5′-Cy3-C**U**G**U**GCAA**U**ATTT**U**GT-3′	55.0	73.0	71.0	+38.0	−46	25.0%	31.3%
	3′-GA**C**A**C**GTTA**U**AAAA**C**A-Cy3-5′	[+4.0]	[+22.0]	[+20.0]				
								
**INV8** **(15)**	5′-Cy3-TT**C**ACAGCCC**U**G**U**GC-3′	58.5 ^b^	70.5	74.5	+26.5	−52	20.0%	60.0%
	3′-AAG**U**GTCGGGA**C**A**C**G-Cy3-5′	[−1.5]	[+10.5]	[+14.5]				
								
**INV9** **(15)**	5′-Cy3-T**U**A**U**ATGCTG**U**TCTC-3′	55.0	58.0	64.0	+21.5	−19	20.0%	33.3%
	3′-AA**U**A**U**ACGACA**A**GAG-Cy3-5′	[+9.5]	[+12.5]	[+18.5]				
								
**INV10 (14)**	5′-Cy3-G**U**G**U**AGTG**U**A**U**ATG-3′	45.5	65.0	64.5	+40.5	−56	28.6%	35.7%
	3′-CA**C**A**U**CACA**U**A**U**AC-Cy3-5′	[+2.0]	[+21.5]	[+21.0]				
								
**OPT6** **(16)**	5′-Cy3-C**U**G**U**G**C**AAC**U**GGT**U**TG-3′	49.0	75.0	75.0	+43.0	−84	31.3%	50.0%
	3′-GA**C**A**C**G**U**TGA**C**CAA**A**C-Cy3-5′	[−9.0]	[+17.0]	[+17.0]				
								
**OPT8** **(15)**	5′-Cy3-TT**C**A**C**AGCCC**U**G**U**GC-3′	38.0 ^b^	77.0	76.5	+55.5	−93	26.7%	60.0%
	3′-AAG**U**G**U**CGGGA**C**A**C**G-Cy3-5′	[−22.0]	[+17.0]	[+16.5]				
								
**OPT9** **(15)**	5′-Cy3-T**U**A**U**A**U**GC**U**G**U**TCTC-3′	29.0 ^b^	65.0	64.0	+54.5	−59	33.3%	33.3%
	3′-AA**U**A**U**A**C**GA**C**A**A**GAG-Cy3-5′	[−16.5]	[+19.5]	[+18.5]				

^a^ Δ*T*_m_ = change in *T*_m_ value relative to corresponding unmodified duplex. *T*_m_s for the corresponding unmodified DNA duplexes are: **DNA1** = 56.0 °C, **DNA2** = 53.0 °C, **DNA3** = 58.5 °C, **DNA4** = 60.5 °C [24], **DNA5** = 57.0 °C, **DNA6** = 58.0 °C, **DNA7** = 51.0 °C, **DNA8** = 60.0 °C, **DNA9** = 45.5 °C, and **DNA10** = 43.5 °C. Thermal denaturation curves were recorded in medium salt buffer ([Na^+^] = 110 mM, [Cl^-^] = 100 mM, pH 7.0 (NaH_2_PO_4_/Na_2_HPO_4_), [EDTA] = 0.2 mM) and each [ON] = 1.0 μM; see main text for definitions of *TA* and Δ*G*${}_{rec}^{310}$. NT = no clear transition observed in *A*_230-280_ range. ND = not determined. For structures of **A**, **C,** and **U**, see Figure 1. ^b^ Broad transition.

**Table 2:** Selected data pertaining to denaturation properties and dsDNA-recognition potential from Spearman’s rank-order correlation analysis of parameter pairs ^a^.

Entry	Parameter Pair	Correlation Coefficient r_s_	*p*-Value
1	probe duplex Δ*T*_m_ × length	−0.169	0.664
2	probe duplex Δ*T*_m_ × GC%	−0.328	0.388
3	probe duplex Δ*T*_m_ × mod%	−0.037	0.924
4	probe duplex Δ*T*_m_ × #mod	−0.091	0.815
5	probe duplex Δ*T*_m_ × stretch	−0.244	0.526
6	5′-ON:cDNA Δ*T*_m_ × mod%	0.774	0.009
7	3′-ON:cDNA Δ*T*_m_ × mod%	0.661	0.037
8	5′-ON:cDNA Δ*T*_m_ × stretch	−0.810	0.005
9	3′-ON:cDNA Δ*T*_m_ × stretch	−0.853	0.002
10	5′-ON:cDNA Δ*T*_m_ × GC%	−0.762	0.010
11	3′-ON:cDNA Δ*T*_m_ × GC%	−0.518	0.125
12	5′-ON:cDNA Δ*T*_m_ × dsDNA *T*_m_	−0.697	0.025
13	3′-ON:cDNA Δ*T*_m_ × dsDNA *T*_m_	−0.515	0.128
14	*TA* × probe duplex Δ*T*_m_	−0.567	0.112
15	*TA* × 5′-ON:cDNA Δ*T*_m_	0.583	0.099
16	*TA* × 3′-ON:cDNA Δ*T*_m_	0.333	0.381
17	*TA* × probe duplex *T*_m_	−0.778	0.014
18	*TA* × GC%	−0.359	0.343
19	*TA* × mod%	0.662	0.052
20	*TA* × #mod	0.822	0.007
21	Δ*G*${}_{rec}^{310}$ × *TA*	−0.814	0.014
22	Δ*G*${}_{rec}^{310}$ × mod%	−0.583	0.129
23	Δ*G*${}_{rec}^{310}$ × #mod	−0.620	0.101

^a^ For the complete dataset, see the Supplementary Materials.

**Table 3:** Rec_100×_ and C_50_ values for recognition of model DNA hairpin targets when using the corresponding Invader probes ^a^.

Probe	Rec_100×_ (%)	C_50_ (µM)
**INV1**	66 ± 1.3	1.3
**INV2**	97 ± 2.8	0.2
**INV3**	60 ± 6.2	2.9
**INV4**	<5	ND
**INV5**	39 ± 7.0	4.1
**INV6**	41 ± 3.4	>10
**INV7**	97 ± 2.6	0.7
**INV8**	96 ± 4.2	0.6
**INV9**	66 ± 1.4	1.5
**INV10**	99 ± 0.0	0.2
**OPT6**	42 ± 3.4	>10
**OPT8**	99 ± 0.4	0.2
**OPT9**	94 ± 8.9	0.6

^a^ Rec_100×_ = level of DNA hairpin recognition using 100-fold molar probe excess (Figure 3). C_50_ values for **INV1**-**INV10** and **OPT6/8/9** were determined from dose–response curves shown in Figure 4 and Figure S24, respectively. “±” = standard deviation. ND = not determined due to low levels of recognition in preliminary screen.

**Table 4:** Selected data from our Spearman’s rank-order correlation analysis pertaining to Invader-mediated recognition of DNA hairpins and chromosomal DNA in FISH assays ^a^.

Entry	Parameter Pair	Correlation Coefficient r_s_	*p*-Value
1	C_50_ × mod%	−0.850	0.008
2	C_50_ × #mod	−0.732	0.039
3	C_50_ × stretch	0.735	0.038
4	C_50_ × *TA*	−0.598	0.156
5	C_50_ × Δ*G*${}_{rec}^{310}$	0.772	0.072
6	C_50_ × 5′-ON:cDNA Δ*T*_m_	−0.782	0.022
7	C_50_ × 3′-ON:cDNA Δ*T*_m_	−0.566	0.144
8	C_50_ × 5′-ON:cDNA Δ*G*^310^	0.604	0.113
9	C_50_ × 3′-ON:cDNA Δ*G*^310^	0.749	0.032
10	C_50_ × probe duplex *T*_m_	0.032	0.945
11	C_50_ × probe duplex Δ*T*_m_	0.010	0.983
12	C_50_ × probe duplex Δ*G*^310^	−0.187	0.723
13	C_50_ × probe duplex ΔΔ*G*^310^	0.138	0.795
14	C_50_ × GC%	0.323	0.435
15	d-FISH × mod%	0.713	0.021
16	d-FISH × C_50_	−0.853	0.007
17	nd-FISH × mod%	0.738	0.015
18	nd-FISH × C_50_	−0.710	0.049
19	d-FISH × #mod	0.558	0.094
20	d-FISH × stretch	−0.711	0.021
21	nd-FISH × #mod	0.547	0.102
22	nd-FISH × stretch	−0.590	0.073
23	nd-FISH × *TA*	0.505	0.165
24	nd-FISH × Δ*G*${}_{rec}^{310}$	−0.583	0.129
25	d-FISH × *TA*	0.274	0.476
26	d-FISH × Δ*G*${}_{rec}^{310}$	−0.319	0.441
27	d-FISH × GC%	−0.099	0.785
28	nd-FISH × GC%	0.191	0.597

^a^ For the complete dataset, see the Supplementary Materials.

**Table 5:** Percent of nuclei presenting a single, punctate signal in d-FISH and nd-FISH assays when incubated with different Invader probes ^a^.

Probe	d-FISH	nd-FISH
**INV1**	0%	0%
**INV2**	~90%	~85%
**INV3**	~40%	~30%
**INV4**	~90%	~90%
**INV5**	0%	0%
**INV6**	~60%	~20%
**INV7**	~60%	~25%
**INV8**	~60%	~25%
**INV9**	~60%	0%
**INV10**	~90%	~90%
**OPT6**	~90%	~85%
**OPT8**	~90%	~75%
**OPT9**	~75%	~25%

^a^ Incubation conditions are as described in Figure 6.

## Data Availability

Not applicable.

## Supplementary Materials

The following are available online at https://www.mdpi.com/article/10.3390/molecules28010127/s1, Figures S1–S24; Tables S1–S11; zipper nomenclature definition; additional discussion; supplementary references [23,24,38].

## Sample Availability

Samples of Invader probes may be requested from the authors.
