# Supplementary material for: Factors Impacting Invader-Mediated Recognition of Double-Stranded DNA
Source: Molecules. 2022 Dec 23;28(1):127. doi: 10.3390/molecules28010127 (PMC9821881; doi:10.3390/molecules28010127)
Supplement: Supplementary file 1 [file molecules-28-00127-s001.zip › molecules-2030208-supplementary.pdf]

Factors impacting Invader-mediated recognition of double-stranded DNA

Caroline P. Shepard, Raymond G. Emehiser, Saswata Karmakar, and Patrick J. Hrdlicka*

Department of Chemistry, University of Idaho, Moscow, Idaho 83844-2343, USA

E-mail: hrdlicka@uidaho.edu

SUPPORTING INFORMATION

TABLE OF CONTENT

Definition of zipper nomenclature S2

Targeted regions within the *DYZ-1* gene on the bovine (*Bos taurus*) Y chromosome (Fig. S1) S3

MALDI-MS data of Invader probe strands (Table S1) S4

MALDI-MS spectra of **INV1**-**INV10** (Figs. S2-S4) S5

Representative thermal denaturation profiles for duplexes entailing **INV1**-**INV10** (Figs. S5 and S6) S8

Changes in Gibbs free energy, enthalpy and entropy for duplexes entailing **INV1**-**INV10** as well as $\Delta G^{310}_{\text{rec}}$, ΔH_{rec}, and $-T\Delta S^{310}_{rec}$ values (Tables S2-S4) S10

Additional discussion regarding Spearman's rank-order correlation analysis of thermodynamic parameters for duplexes entailing **INV1**-**INV10** (Table S5) S13

Sequences and intramolecular T_ms of DNA hairpins used in present study (Table S6) S15

Recognition of mixed-sequence model DNA hairpin targets using **INV1**-**INV10** and shorter incubation times (Figs. S7-S10 and Table S7) S17

Dose-response experiments for recognition of DNA hairpins using **INV1**-**INV10**

following 15 h of incubation (Figs. S11-S13) S23
Representative images from FISH experiments using **INV1-INV10** (Figs. S14-S17) S26
Representative images from FISH experiments after DNase I pre-treatment (Fig. S18) S30
Representative images from FISH experiments after RNase A or Proteinase K pre-treatment (Fig. S19) S31
MALDI-MS data of optimized Invader probe strands (Table S8) S32
MALDI-MS spectra of **OPT6**, **OPT8** and **OPT9** (Fig. S20) S33
Representative thermal denaturation profiles for duplexes entailing optimized Invader probe strands (Fig. S21) S34
Changes in Gibbs free energy, enthalpy and entropy for duplexes entailing **OPT6/8/9** as well as $\Delta G^{310}_{\text{rec}}$, ΔH_{rec}, and $-T\Delta S^{310}_{rec}$ values (Tables S9-S11) S35
Representative gel electrophoretograms from initial recognition screen using **OPT6**, **OPT8**, and **OPT9** (Fig. S22) S37
Dose-response experiments for recognition of DNA hairpins using **OPT6/8/9** (Figs. S23 and S24) S38
Supplementary references S40

Definition of zipper nomenclature. The following nomenclature is used to describe the relative arrangement between two 2′-*O*-(pyren-1-yl)methyl-RNA monomers on opposing strands in an Invader probe. The number *n* describes the distance measured in number of base-pairs and has a positive value if a monomer is shifted toward the 5′-side of its

strand relative to a second reference monomer on the other strand. Conversely, n has a negative value if a monomer is shifted toward the 3′-side of its strand relative to a second reference monomer on the other strand.

GGGATCTCAGCCCTGTGGTCTGGGAACTGGGAAACCGGCTTGAGTATGTGTGCTG[1]**TTATCAGCACTGTGC**CCTGGCAACTCTG
[2]**ATACTGGTTTGTGTTC**ATGTGTGTGTGTGTGTGTGTGTGTGTTGCTGTTCTC[4]AGCCCTGTGCCCTGGCAACTGTGCAACCAGTATC
TGTATGCCTGTGTGTGTGTGTGTGTGTGTGTGTGTGTGTGTGTGCTGTTCTCAACCCATTGCCCTGGCAACTGTTCAACCAGTTT
GTGTATATGTGTGTGTAGATGTGTGTGCCATCCTGAGCC[3]**TTGTGCCCTGGCAAC**TGGGGAAACGGTGTGTGTGTGTTGTGTGTCT
GTGTGTGTGCT[5]**GATTTCAGCCATGTGC**CCATCTAA[6]**CTGTGCAACTGGTTTG**TGTGTGTGTGCACGCGATTCTCACCTCTGTGTCC
TGGCAACTGTGTAACCGTTTGTGTGTGTGAGTGTGTGTAAGTGTGTGCTCTTTTCAGCCCTGTTTCCTAGAAACTGTGGAACCGGTT
GGTGTGTGTGTGTGTCTGTGTGTGTGTGTGCCATTCTC[4]AGCCCTGTGCCCTGGCAA[7]**CTGTGCAATATTTTGT**CGTGTGTGTGTGTG
TGTGTATTTGTGTGTGCAATTCACAGCCCTGTTCCCTGGCAACTGTGCAAGCAGATTGTTGCGTATGTTTCTGTGTGTGTGTGTGTG
TGTGTGTGTGTGTATGTGCTGTTCTC[4]AGCCCTGTGCCCTGGCAACTGTGAAACCGGTTTGTATGTGTGTGTGTGTTTGTGTGTGCCA
[8]**TTCAC**[4]**AGCCCTGTGC**CCTGGCAACTGTGCAAGCAGTTTGTGTGTGCATGTGTCTGTGTGTGTATGTGTCTGTGTGTGCATGTGTCT
GTGTGTG[9]**TTATATGCTGTTCTC**[4]AGCCCTGTGCCCTGGCAACTGAGAAACCGGTTGTGTGTGTGTGTGTGTGTGTGTGTGTGTGTGC
CAGTTTCAGCCCTGTGCCTTGGTAACTGTGCAAGTGGTTTGTGTGTGTGT[10]**GTGTAGTGTATATG**TGTGTGTGTGGTTTGACCAGTT
TTCAGCCCTGTGCCTTAGTAACTGTGTAACTGGTGTGTGTGTGTGTGTGTGTGTGTGTGCTCTTCTC[4]AGCCCTGTGCCCTGTTAACT
GTGCAAGCGGTTTGTCTGTGTATGTGAGTGGGTGCTGTTCTCATGCCTGTGCACTGG

Figure S1. Regions within the *DYZ-1* satellite gene on the bovine (*Bos taurus*) Y chromosome that are targeted by the different Invader probes [S1]. The target sequence for **INV4** (highlighted in grey) is present six times within the tandem repeat (~6 x 10^4 tandem repeats); all other target sequences are present only once (highlighted in green). Target region for **INV8** overlaps partially with one of the **INV4** target regions.

Table S1. MALDI-MS of individual Invader probe strands.[a]

ON	Sequence	Obs. *m/z* [M+H]$^+$	Calc. *m/z* [M+H]$^+$
**INV1u**	5'-Cy3-T**U**ATCAGCAC**U**G**U**GC-3'	5700	5697
**INV1d**	3'- AA**U**AGTCGTGA**C**A**C**G-Cy3-5'	5785	5783
**INV2u**	5'-Cy3-A**U**AC**U**GGTTTG**U**G**U**TC-3'	6266[b]	6264
**INV2d**	3'- TA**U**GA**C**CAAACA**C**A**A**G-Cy3-5'	6282[b]	6279
**INV3u**	5'-Cy3-T**U**G**U**GCCCTGGC**A**AC-3'	5714	5712
**INV3d**	3'- AA**C**A**C**GGGACCGT**U**G-Cy3-5'	5786	5783
**INV4u**	5′-Cy3-**A**GCCC**U**GTGCC**C**TG-3'	5400[c]	5398
**INV4d**	3′- T**C**GGGA**C**ACGGG**A**C-Cy3-5'	5511[c]	5510
**INV5u**	5'-Cy3-G**A**TTTCAGCCA**U**G**U**GC-3'	6043	6040
**INV5d**	3'- CT**A**AAGTCGGTA**C**A**C**G-Cy3-5'	6089	6086
**INV6u**	5'-Cy3-C**U**G**U**GCAACTGGT**U**TG-3'	6064	6057
**INV6d**	3'- GA**C**A**C**GTTGACCAA**A**C-Cy3-5'	6057	6055
**INV7u**	5'-Cy3-C**U**G**U**GCAA**U**ATTT**U**GT-3'	6249	6247
**INV7d**	3'- GA**C**A**C**GTTA**T**AAAA**C**A-Cy3-5'	6296	6295
**INV8u**	5'-Cy3-TT**C**ACAGCCC**U**G**U**GC-3'	5673	5673
**INV8d**	3'- AAG**U**GTCGGGA**C**A**C**G-Cy3-5'	5828	5824
**INV9u**	5'-Cy3-T**U**A**U**ATGCTG**U**TCTC-3'	5680	5678
**INV9d**	3'- AA**U**A**U**ACGACA**A**GAG-Cy3-5'	5789	5787
**INV10u**	5'-Cy3-G**U**G**U**AGTG**U**A**U**ATG-3'	5721[b]	5720
**INV10d**	3'- CA**C**A**U**CACA**U**A**U**AC-Cy3-5'	5562[b]	5561

[a] Individual strands are arbitrarily denoted up (u) or down (d).

[b]MALDI-MS data previously reported in reference S2.

[c]MALDI-MS data previously reported in reference S3.

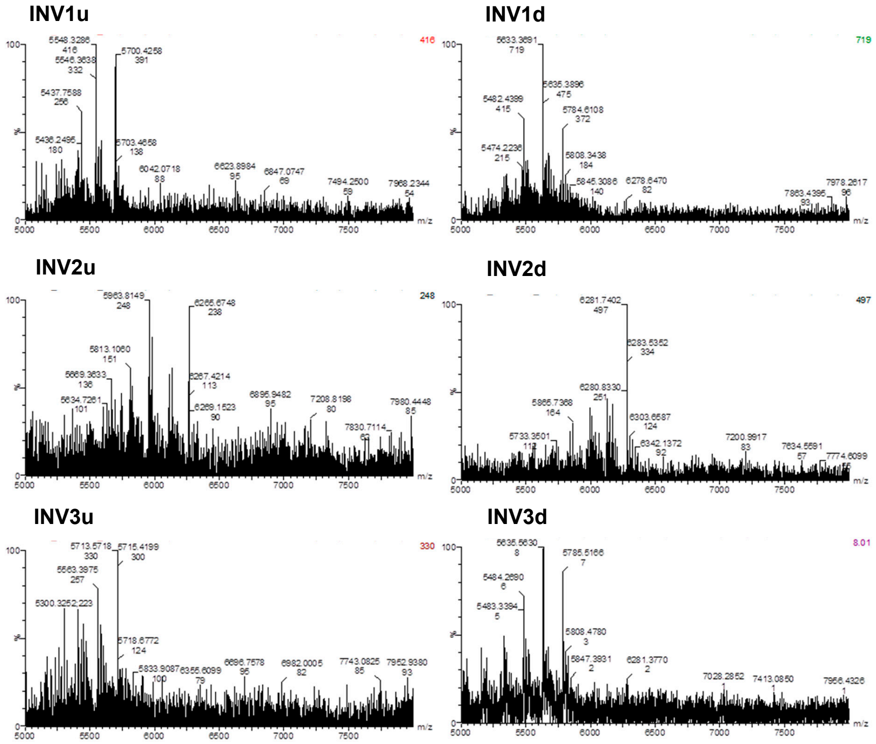

Figure S2. MALDI-MS spectra of individual Invader probe strands **INV1-INV3**.

Commented [M1]: To avoid any errors during position changes, please provide the combined image instead of editable pieces in the figure. Same as follows.

Commented [HP(2R1]: Will be provided

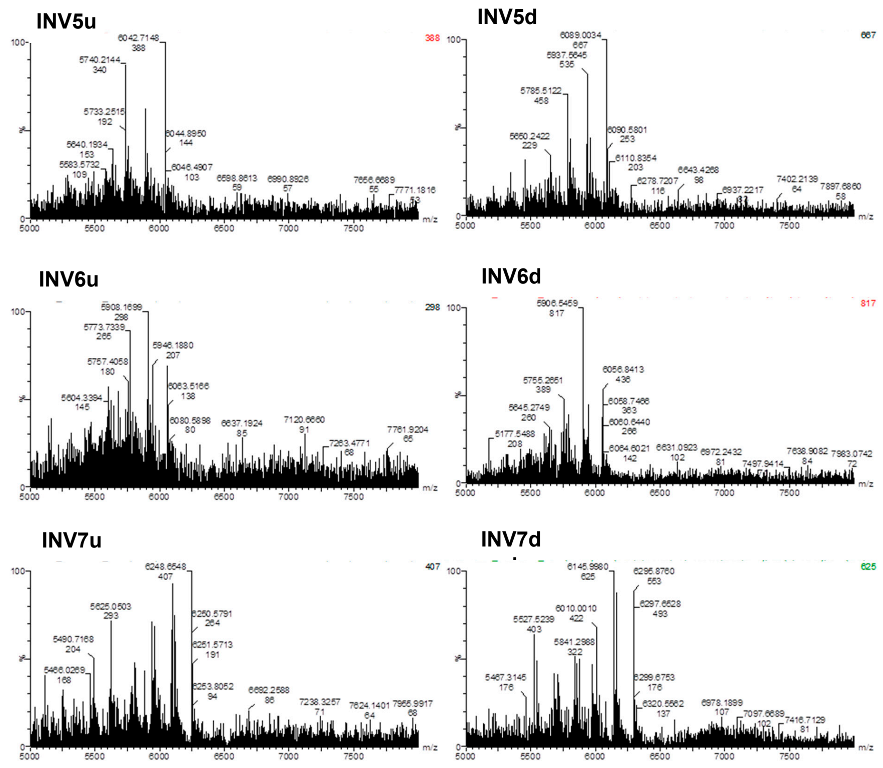

Figure S3. MALDI-MS spectra of individual Invader probe strands **INV5-INV7**.

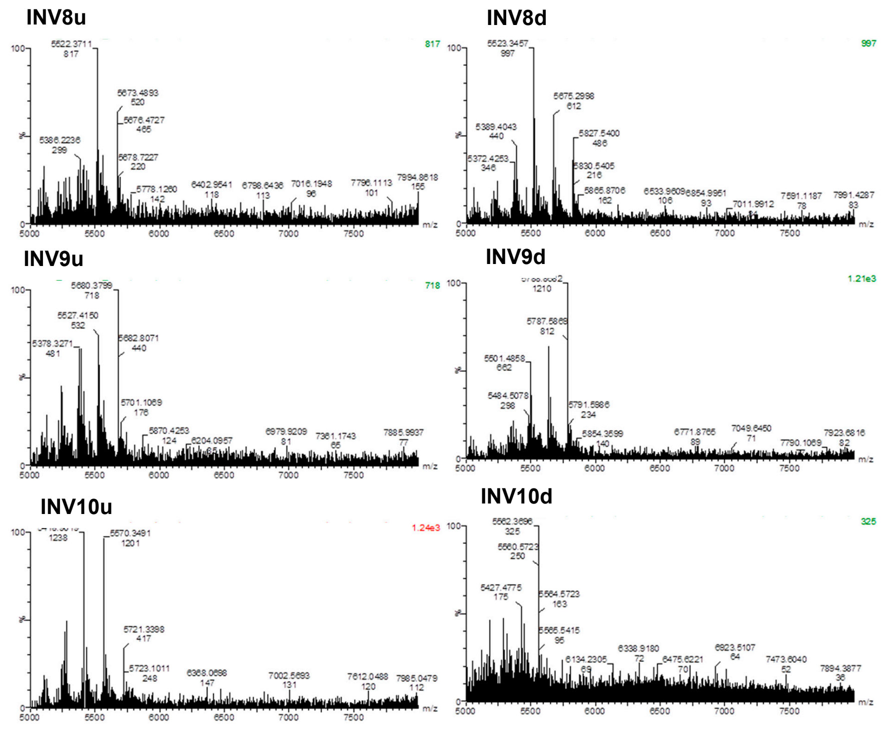

Figure S4. MALDI-MS spectra of individual Invader probe strands **INV8-INV10**.

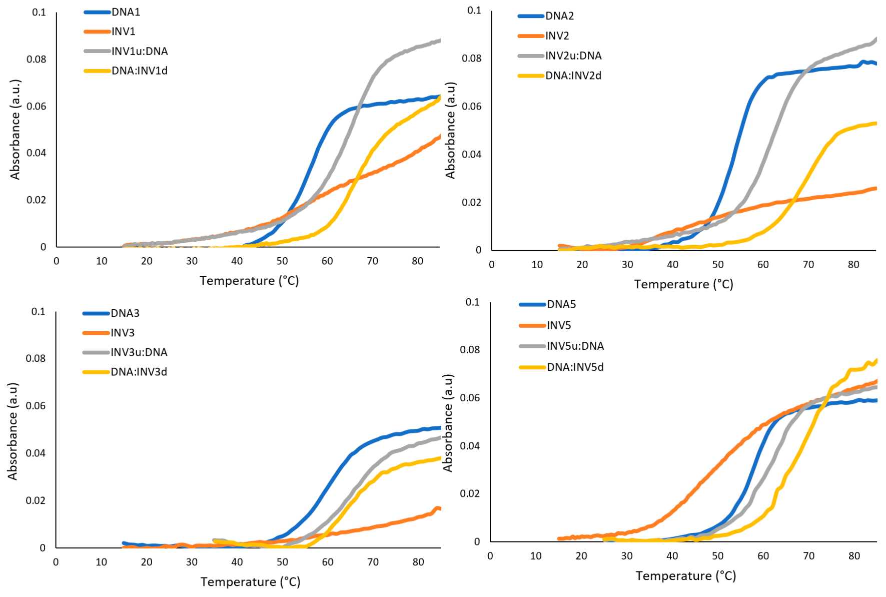

Figure S5. Representative thermal denaturation profiles for Invader probes **INV1**-**INV3** and **INV5**, the corresponding duplexes between individual probe strands and cDNA, and the unmodified reference DNA duplexes (**DNA1**-**DNA3** and **DNA5**).

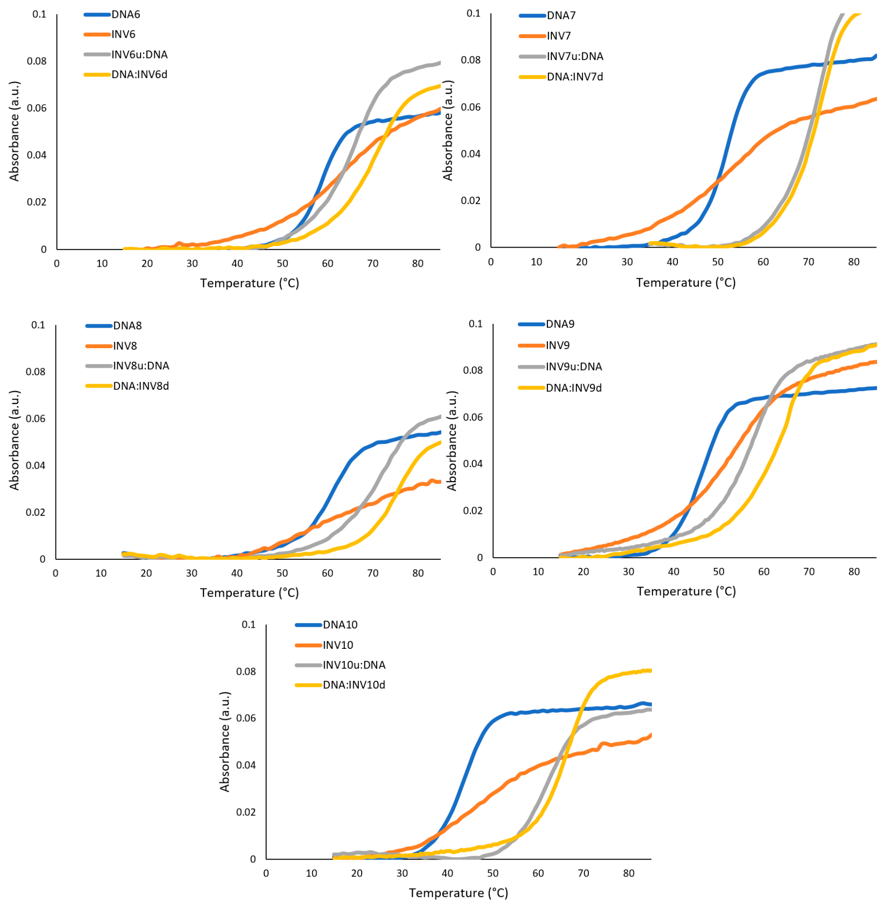

Figure S6. Representative thermal denaturation curves for Invader probes **INV6-INV10** and the corresponding duplexes between individual probe strands and cDNA, and unmodified reference DNA duplexes.

Table S2. Change in Gibbs free energy (ΔG^{310}) upon formation of double-stranded probes and duplexes between individual probe strands and cDNA. Also shown is the calculated change in reaction free energy upon Invader-mediated recognition of isosequential dsDNA targets ($\Delta G^{310}_{\mathrm{rec}}$).[a.]

Probe	Sequence	ΔG^{310} [$\Delta\Delta G^{310}$] (kJ/mol)			ΔG^{310}_{rec} (kJ/mol)
		Probe duplex	5'ON: cDNA	3'ON: cDNA	
**INV1**	5'-Cy3-T**U**ATCAGCAC**U**G**U**GC-3'<br>3'- AA**U**AGTCGTGA**C**A**C**G-Cy3-5'	ND	-72<br>[-8]	-45<br>[+19]	ND
**INV2**	5'-Cy3-A**U**AC**U**GGTTTG**U**G**U**TC-3'<br>3'- TA**U**GA**C**CAAACA**C**A**A**G-Cy3-5'	-39<br>[+25]	-72<br>[-8]	-81<br>[-17]	-50
**INV3**	5'-Cy3-T**U**G**U**GCCCTGGC**A**AC-3'<br>3'- AA**C**A**C**GGGACCGT**U**G-Cy3-5'	ND	-66<br>[-3]	-69<br>[-6]	ND
**INV4**	5'-Cy3-**A**GCCC**U**GTGCC**C**TG-3'<br>3'- T**C**GGGA**C**ACGGG**A**C-Cy3-5'	-65<br>[+7]	-54<br>[+18]	-90<br>[-18]	-7
**INV5**	5'-Cy3-G**A**TTTCAGCCA**U**G**U**GC-3'<br>3'- CT**A**AAGTCGGTA**C**A**C**G-Cy3-5'	-46<br>[+25]	-68<br>[+3]	-78<br>[-7]	-29
**INV6**	5'-Cy3-C**U**G**U**GCAACTGGT**U**TG-3'<br>3'- GA**C**A**C**GTTGACCAA**A**C-Cy3-5'	-58<br>[+14]	-73<br>[-1]	-79<br>[-7]	-22
**INV7**	5'-Cy3-C**U**G**U**GCAA**U**ATTT**U**GT-3'<br>3'- GA**C**A**C**GTTA**T**AAAA**C**A-Cy3-5'	-50<br>[+12]	-75<br>[-13]	-83<br>[-21]	-46
**INV8**	5'-Cy3-TT**C**ACAGCCC**U**G**U**GC-3'<br>3'- AAG**U**GTCGGGA**C**A**C**G-Cy3-5'	-50<br>[+21]	-74<br>[-3]	-99<br>[-28]	-52
**INV9**	5'-Cy3-T**U**A**U**ATGCTG**U**TCTC-3'<br>3'- AA**U**A**U**ACGACA**A**GAG-Cy3-5'	-53<br>[-1]	-62<br>[-10]	-62<br>[-10]	-19
**INV10**	5'-Cy3-G**U**G**U**AGTG**U**A**U**ATG-3'<br>3'- CA**C**A**U**CACA**U**A**U**AC-Cy3-5'	-47<br>[+2]	-70<br>[-21]	-82<br>[-33]	-56

[a]$\Delta\Delta G^{310}$ is measured relative to the corresponding unmodified DNA duplexes (**DNA1** = -64 kJ/mol, **DNA2** = -64 kJ/mol, **DNA3** = -63 kJ/mol, **DNA4** = -72 kJ/mol, **DNA5** = -71 kJ/mol, **DNA6** = -72 kJ/mol, **DNA7** = -62 kJ/mol, **DNA8** = -71 kJ/mol, **DNA9** = -52 kJ/mol, and **DNA10** = -49 kJ/mol). ND = not determined as broad thermal denaturation transitions precluded confident baseline fitting. ΔG^{310}_{rec} = ΔG^{310} (5'-ON:cDNA) + ΔG^{310} (3'-ON:cDNA) - ΔG^{310} (probe duplex) – ΔG^{310} (dsDNA).

Table S3. Change in enthalpy (ΔH) upon formation of double-stranded probes and duplexes between individual probe strands and cDNA. Also shown is the calculated change in reaction enthalpy upon Invader-mediated recognition of isosequential dsDNA targets (ΔH_{rec}).[a]

Probe	Sequence	ΔH[ΔΔH] (kJ/mol)			ΔH_{rec} (kJ/mol)
		Probe duplex	5'ON: cDNA	3'ON: cDNA	
**INV1**	5'-Cy3-T**U**ATCAGCAC**U**G**U**GC-3'<br>3'- AA**U**AGTCGTGA**C**A**C**G-Cy3-5'	ND	-414<br>[+40]	-345<br>[+109]	ND
**INV2**	5'-Cy3-A**U**AC**U**GGTTTG**U**G**U**TC-3'<br>3'- TA**U**GA**C**CAAACA**C**A**A**G-Cy3-5'	-159<br>[+372]	-458<br>[+73]	-476<br>[+55]	-244
**INV3**	5'-Cy3-T**U**G**U**GCCCTGGC**A**AC-3'<br>3'- AA**C**A**C**GGGACCGT**U**G-Cy3-5'	ND	-358<br>[+8]	-393<br>[-27]	ND
**INV4**	5'-Cy3-**A**GCCC**U**GTGCC**C**TG-3'<br>3'- T**C**GGGA**C**ACGGG**A**C-Cy3-5'	-295<br>[+174]	-203<br>[+266]	-472<br>[-3]	+89
**INV5**	5'-Cy3-G**A**TTTCAGCCA**U**G**U**GC-3'<br>3'- CT**A**AAGTCGGTA**C**A**C**G-Cy3-5'	-240<br>[+286]	-422<br>[+104]	-448<br>[+78]	-104
**INV6**	5'-Cy3-C**U**G**U**GCAACTGGT**U**TG-3'<br>3'- GA**C**A**C**GTTGACCAA**A**C-Cy3-5'	-267<br>[+253]	-422<br>[+98]	-441<br>[+79]	-76
**INV7**	5'-Cy3-C**U**G**U**GCAA**U**ATTT**U**GT-3'<br>3'- GA**C**A**C**GTTA**T**AAAA**C**A-Cy3-5'	-242<br>[+285]	-418<br>[+109]	-474<br>[+53]	-123
**INV8**	5'-Cy3-TT**C**ACAGCCC**U**G**U**GC-3'<br>3'- AAG**U**GTCGGGA**C**A**C**G-Cy3-5'	-209<br>[+262]	-373<br>[+98]	-567<br>[-96]	-260
**INV9**	5'-Cy3-T**U**A**U**ATGCTG**U**TCTC-3'<br>3'- AA**U**A**U**ACGACA**A**GAG-Cy3-5'	-275<br>[+188]	-386<br>[+77]	-329<br>[+134]	+23
**INV10**	5'-Cy3-G**U**G**U**AGTG**U**A**U**ATG-3'<br>3'- CA**C**A**U**CACA**U**A**U**AC-Cy3-5'	-240<br>[+275]	-425<br>[+90]	-488<br>[+27]	-158

[a]ΔΔH is measured relative to the corresponding unmodified DNA duplex (**DNA1** = -454 kJ/mol, **DNA2** = -531 kJ/mol, **DNA3** = -366 kJ/mol, **DNA4** = -469 kJ/mol, **DNA5** = -526 kJ/mol, **DNA6** = -520 kJ/mol, **DNA7** = -527 kJ/mol, **DNA8** = -471 kJ/mol, **DNA9** = -463 kJ/mol, and **DNA10** = -515 kJ/mol). ND = not determined as broad thermal denaturation transitions precluded confident baseline fitting. ΔH_{rec} = ΔH (5'-ON:cDNA) + ΔH (3'-ON:cDNA) – ΔH (probe duplex) – ΔH (dsDNA).

Table S4. Change in entropy ($-T\Delta S^{310}$) upon formation of double-stranded probes and duplexes between individual probe strands and cDNA. Also shown is the calculated change in reaction entropy upon Invader-mediated recognition of isosequential dsDNA targets ($-T\Delta S^{310}_{rec}$).[a]

Probe	Sequence	$-T\Delta S^{310}$ [$\Delta(T\Delta S^{310})$] (kJ/mol) Probe duplex	5'ON: cDNA	3'ON: cDNA	$-T\Delta S^{310}_{rec}$ (kJ/mol)
**INV1**	5'-Cy3-T**U**ATCAGCAC**U**G**U**GC-3'<br>3'- AA**U**AGTCGTGA**C**A**C**G-Cy3-5'	ND	341<br>[-49]	300<br>[-90]	ND
**INV2**	5'-Cy3-A**U**AC**U**GGTTTG**U**G**U**TC-3'<br>3'- TA**U**GA**C**CAAACA**C**A**A**G-Cy3-5'	119<br>[-347]	386<br>[-80]	394<br>[-72]	195
**INV3**	5'-Cy3-T**U**G**U**GCCCTGGC**A**AC-3'<br>3'- AA**C**A**C**GGGACCGT**U**G-Cy3-5'	ND	292<br>[-11]	324<br>[+21]	ND
**INV4**	5'-Cy3-**A**GCCC**U**GTGCC**C**TG-3'<br>3'- T**C**GGGA**C**ACGGG**A**C-Cy3-5'	229<br>[-168]	148<br>[-249]	382<br>[-15]	-96
**INV5**	5'-Cy3-G**A**TTTCAGCCA**U**G**U**GC-3'<br>3'- CT**A**AAGTCGGTA**C**A**C**G-Cy3-5'	194<br>[-261]	354<br>[-101]	370<br>[-85]	75
**INV6**	5'-Cy3-C**U**G**U**GCAACTGGT**U**TG-3'<br>3'- GA**C**A**C**GTTGACCAA**A**C-Cy3-5'	209<br>[-239]	348<br>[-100]	362<br>[-86]	53
**INV7**	5'-Cy3-C**U**G**U**GCAA**U**ATTT**U**GT-3'<br>3'- GA**C**A**C**GTTA**T**AAAA**C**A-Cy3-5'	192<br>[-273]	343<br>[-122]	390<br>[-75]	76
**INV8**	5'-Cy3-TT**C**ACAGCCC**U**G**U**GC-3'<br>3'- AAG**U**GTCGGGA**C**A**C**G-Cy3-5'	160<br>[-240]	299<br>[-101]	467<br>[+67]	206
**INV9**	5'-Cy3-T**U**A**U**ATGCTG**U**TCTC-3'<br>3'- AA**U**A**U**ACGACA**A**GAG-Cy3-5'	222<br>[-189]	324<br>[-87]	262<br>[-149]	-47
**INV10**	5'-Cy3-G**U**G**U**AGTG**U**A**U**ATG-3'<br>3'- CA**C**A**U**CACA**U**A**U**AC-Cy3-5'	193<br>[-272]	355<br>[-110]	406<br>[-59]	103

[a]$\Delta(T\Delta S^{310})$ is measured relative to the corresponding unmodified DNA duplex (**DNA1** = 390 kJ/mol, **DNA2** = 466 kJ/mol, **DNA3** = 303 kJ/mol, **DNA4** = 397 kJ/mol, **DNA5** = 455 kJ/mol, **DNA6** = 448 kJ/mol, **DNA7** = 465 kJ/mol, **DNA8** = 400 kJ/mol, **DNA9** = 411 kJ/mol, and **DNA10** = 465 kJ/mol). ND = not determined as broad thermal denaturation transitions precluded confident baseline fitting. $-T^{310}\Delta S_{rec} = T^{310}\Delta S$ (5'-ON:cDNA) + $T^{310}\Delta S$ (3'-ON:cDNA) – $T^{310}\Delta S$ (probe duplex) – $T^{310}\Delta S$ (dsDNA target).

Additional discussion regarding Spearman rank-order correlation analysis of thermodynamic parameters. Our Spearman's rank-order correlation analysis of the dataset indicates that there is a lack of correlation (×) between the $\Delta\Delta G^{310}$ values of the Invader probes and their GC-content or modification density, though there is a correlation with the "longest unmodified stretch" metric that is approaching significance (entries 1-3, Table S5). This reinforces the conclusions from the Spearman rank-order correlation analysis of the probe duplex ΔT_m parameter (entries 1-5, Table 2), suggesting that introduction of the energetic hotspots does not significantly change the stability of the probe in a straightforward manner.

In contrast, negative correlations approaching significance between the $\Delta\Delta G^{310}$ values of probe-cDNA duplexes and modification densities or number of modifications were observed (entries 4-7, Table S5). A positive correlation approaching significance with the longest unmodified stretch metric was also observed (entries 8 and 9, Table S5). In concert, and just as with the T_m-based discussion in the main manuscript, this suggests that densely modified probe strands with short unmodified stretches, result in the most stable probe-target duplexes. Unlike the corresponding T_m-based discussion, it is less clear if there is a correlation between the $\Delta\Delta G^{310}$ values of probe-cDNA duplexes and GC-content or the T_m of the corresponding unmodified DNA duplex (entries 10-13, Table S5).

Table S5. Selected data from Spearman's rank-order correlation analysis pertaining to thermodynamic parameters.[a]

Entry	Parameter pair	Correlation coefficient r_s	ϱ value
1	probe duplex $\Delta\Delta G^{310}$ × GC%	0.321	0.438
2	probe duplex $\Delta\Delta G^{310}$ × mod%	-0.325	0.432
3	probe duplex $\Delta\Delta G^{310}$ × stretch	0.573	0.138
4	5'-ON:cDNA $\Delta\Delta G^{310}$ × mod%	-0.600	0.067
5	3'-ON:cDNA $\Delta\Delta G^{310}$ × mod%	-0.663	0.037
6	5'-ON:cDNA $\Delta\Delta G^{310}$ × #mod	-0.648	0.043
7	3'-ON:cDNA $\Delta\Delta G^{310}$ × #mod	-0.572	0.084
8	5'-ON:cDNA $\Delta\Delta G^{310}$ × stretch	0.582	0.078
9	3'-ON:cDNA $\Delta\Delta G^{310}$ × stretch	0.763	0.010
10	5'-ON:cDNA $\Delta\Delta G^{310}$ × GC%	0.850	0.002
11	3'-ON:cDNA $\Delta\Delta G^{310}$ × GC%	0.202	0.576
12	5'-ON:cDNA $\Delta\Delta G^{310}$ × dsDNA T_m	0.863	0.001
13	3'-ON:cDNA $\Delta\Delta G^{310}$ × dsDNA T_m	0.207	0.567

a) For the complete dataset, see the Supplementary Materials.

Table S6. Sequences and intramolecular T_ms of DNA hairpins.[a]

Hairpin	Sequence	T_m (°C)
**DH1**	5'-TTA TCA GCA CTG TGC<br>3'-AAT AGT CGT GAC ACG<br>(loop: T_{10})	76.0
**DH2**	5'-ATA CTG GTT TGT GTT C<br>3'-TAT GAC CAA ACA CAA G<br>(loop: T_{10})	72.0
**DH3**	5'-TTG TGC CCT GGC AAC<br>3'-AAC ACG GGA CCG TTG<br>(loop: T_{10})	81.5
**DH4**	5'-AGC CCT GTG CCC TG<br>3'-TCG GGA CAC GGG AC<br>(loop: T_{10})	82.0
**DH5**	5'-GAT TTC AGC CAT GTG C<br>3'-CTA AAG TCG GTA CAC G<br>(loop: T_{10})	76.0
**DH6**	5'-CTG TGC AAC TGG TTT G<br>3'-GAC ACG TTG ACC AAA C<br>(loop: T_{10})	75.0
**DH7**	5'-CTG TGC AAT ATT TTG T<br>3'-GAC ACG TTA TAA AAC A<br>(loop: T_{10})	68.0
**DH8**	5'-TTC ACA GCC CTG TGC<br>3'-AAG TGT CGG GAC ACG<br>(loop: T_{10})	80.5
**DH9**	5'-TTA TAT GCT GTT CTC<br>3'-AAT ATA CGA CAA GAG<br>(loop: T_{10})	67.0
**DH10**	5'-GTG TAG TGT ATA TG<br>3'-CAC ATC ACA TAT AC<br>(loop: T_{10})	62.0

**DH2m**	5'-ATA CTG GAT TGT GTT C T_{10}<br>3'-TAT GAC CTA ACA CAA G	nd

Table S6 - continued. Sequences and intramolecular T_ms of DNA hairpins.[a]

Hairpin	Sequence	T_m (°C)
**DH2mm**	5'-ATA CAG GTT TGA GTT C<br>3'-TAT GTC CAA ACT CAA G	nd
**DH6m**	5'-CTG TGC ATC TGG TTT G<br>3'-GAC ACG TAG ACC AAA C	nd
**DH6mm**	5'-CTG TGG AAC TCG TTT G<br>3'-GAC ACC TTG AGC AAA C	nd
**DH8m**	5'-TTC ACA GGC CTG TGC<br>3'-AAG TGT CCG GAC ACG	nd
**DH8mm**	5'-TTC AGA GCC GTG TGC<br>3'-AAG TCT CGG CAC ACG	nd
**DH9m**	5'-TTA TAT GGT GTT CTC<br>3'-AAT ATA CCA CAA GAG	nd
**DH9mm**	5'-TTA TTT GCT CTT CTC<br>3'-AAT AAA CGA GAA GAG	nd
**DH10m**	5'-GTG TAG AGT ATA TG<br>3'-CAC ATC TCA TAT AC	nd
**DH10mm**	5'-GTG TTG TGT TTA TG<br>3'-CAC AAC ACA AAT AC	nd

[a] T_ms were determined as described in Table 1.

Recognition of mixed-sequence model DNA hairpin targets – preliminary experiments using shorter incubation times. Screens in which a 100-fold molar excess of each Invader probe was incubated with the corresponding DNA hairpin target for 2.5 hours at 37 °C, i.e., a shorter period relative to the experiments discussed in the main manuscript, indicated that dsDNA-recognition is incomplete (compare Fig. S7 vs Fig. 3). Thus, while **INV2** and **INV10** displayed high levels of recognition (>80%), **INV7**-**INV9** resulted in moderately levels of recognition (30-60%), whilst **INV1** and **INV3**-**INV6** resulted in no or low levels of recognition (<25%) (Fig. S7 and Table S7). Although some high-affinity Invader probes allow for fast (but still incomplete) recognition of model DNA hairpin targets following 2.5 hours of incubation, most probes require longer incubation times for maximal recognition. This is further underscored upon comparison of C_{50} values following 2.5 or 15 hours of incubation (compare Tables S7 and 3, respectively). For results from dose-response experiments following 2.5 h of incubation, see Figs. S8-S10 and Table S7.

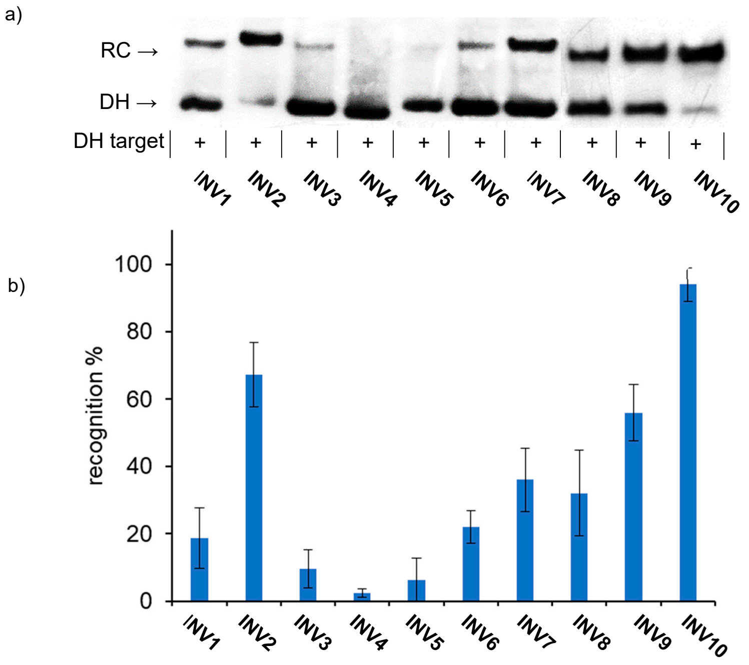

Figure S7. a) Representative gel electrophoretograms from recognition experiments in which a 100-fold molar excess of Invader probes **INV1-INV10** was incubated with their respective DNA hairpin targets **DH1-DH10**. b) Histograms depict averaged results from at least three recognition experiments with error bars representing standard deviation. RC = recognition complex. DH = DNA hairpin. DIG-labeled DNA hairpins **DH1-DH10** (34.4 nM, sequences shown in Table S6) were incubated with the corresponding Invader probe in HEPES buffer (50 mM HEPES, 100 mM NaCl, 5 mM $MgCl_2$, pH 7.2, 10% sucrose, 1.44 mM spermine tetrahyrdochloride) at 37 °C for 2.5 h. Incubation mixtures were resolved on 12% non-denaturing TBE-PAGE slabs (~70 V, ~4 °C, ~1.5 h).

Commented [M3]: To avoid any errors during position changes, please provide the combined image instead of editable pieces in the figure.

Commented [HP(4R3]: Will be provided

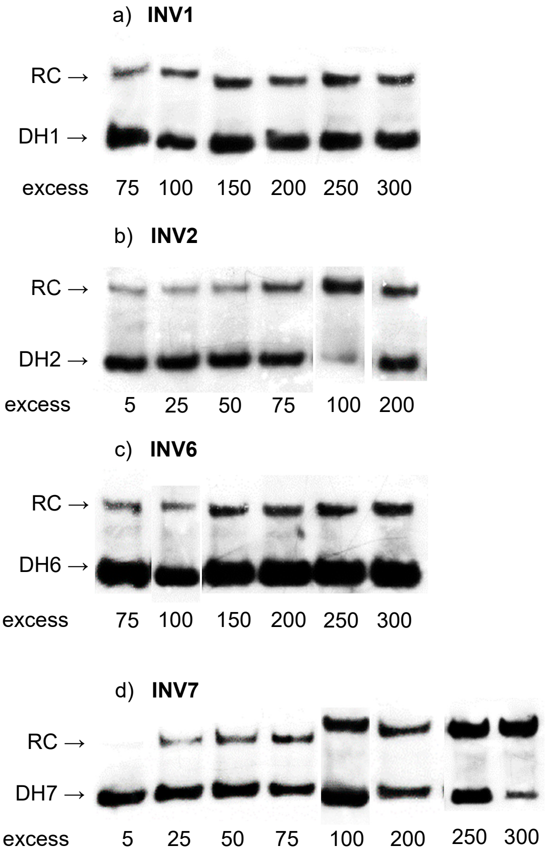

Figure S8. Dose-response experiments. Representative electrophoretograms for recognition of model DNA hairpin targets (34.4 nM) using different concentrations of the corresponding Invader probes a) **INV1**, b) **INV2**, c) **INV6**, and d) **INV7** following incubation at 37 °C for 2.5 h. Experimental conditions are as specified in Figure S7. Corresponding dose-response curves are shown in Figure S10.

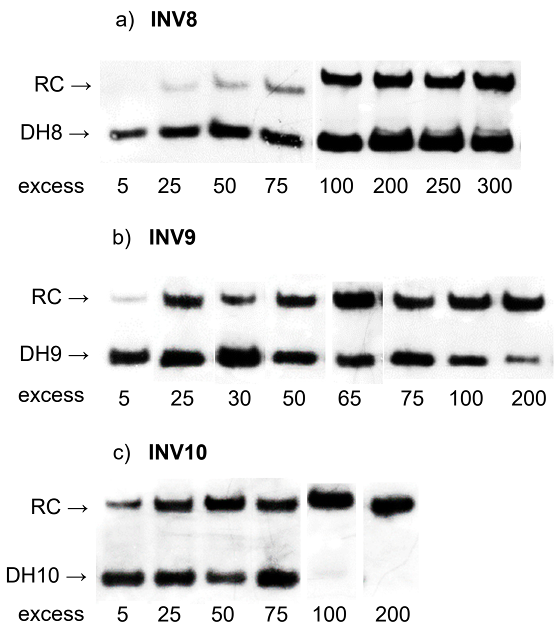

Figure S9. Dose-response experiments. Representative electrophoretograms for recognition of model DNA hairpin targets (34.4 nM) using different concentrations of the corresponding Invader probes a) **INV8**, b) **INV9**, and c) **INV10** following incubation at 37 °C for 2.5 h.. Experimental conditions are as specified in Figure S7. Corresponding dose-response curves are shown in Figure S10.

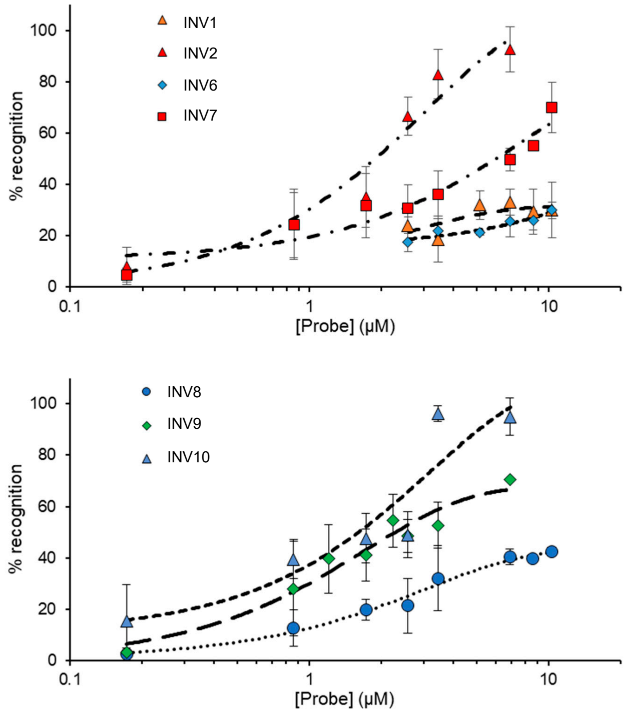

Figure S10. Dose-response curves for **INV1**, **INV2**, **INV6**, and **INV7** (upper panel), and **INV8**-**INV10** (lower panel) following incubation at 37 °C for 2.5 h. Experimental conditions are as described in Figure S7, except for variable probe concentrations.

Table S7. C_{50} values for recognition of model DNA hairpin targets using the corresponding Invader probes following incubation at 37 °C for 2.5 h.[a]

Probe	C_{50} (μM)	Rec_{100X} (%)
**INV1**	>10	18 ± 9
**INV2**	1.9	82 ± 10
**INV3**	ND	9 ± 6
**INV4**	ND	2 ± 1
**INV5**	ND	6 ± 7
**INV6**	>10	22 ± 5
**INV7**	6.0	36 ± 9
**INV8**	>10	32 ± 13
**INV9**	2.2	56 ± 8
**INV10**	1.6	93 ± 5

[a] Rec_{100X} = level of DNA hairpin recognition using 100-fold molar probe excess. C_{50} values were determined from dose-response curves shown in Figure S10. ND = Not determined due to low levels of recognition in the initial screen (Fig. S7).

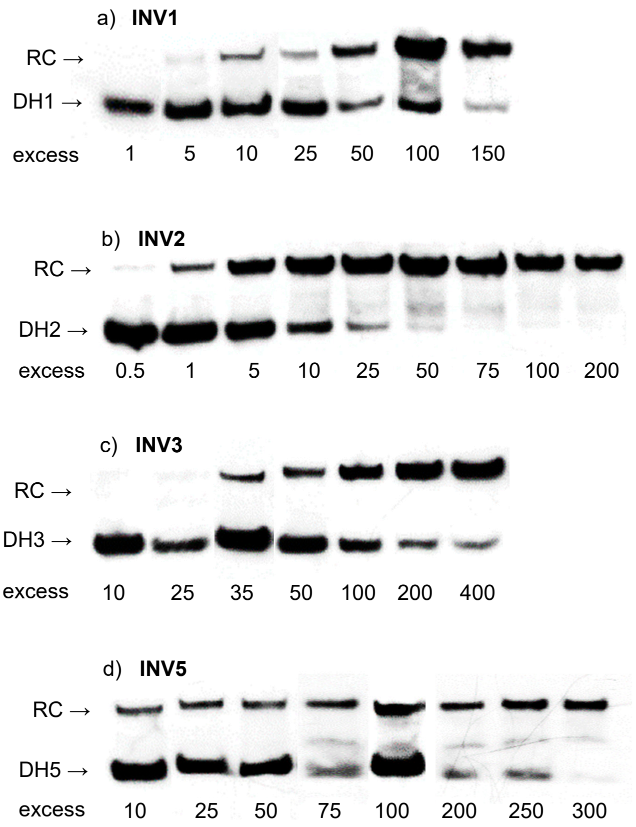

Figure S11. Dose-response experiments. Representative electrophoretograms for recognition of model DNA hairpin targets (34.4 nM) using different concentrations of the corresponding Invader probes a) **INV1**, b) **INV2**, c) **INV3**, and d) **INV5** following incubation at 37 °C for 15 h. Experimental conditions are as specified in Figure 3.

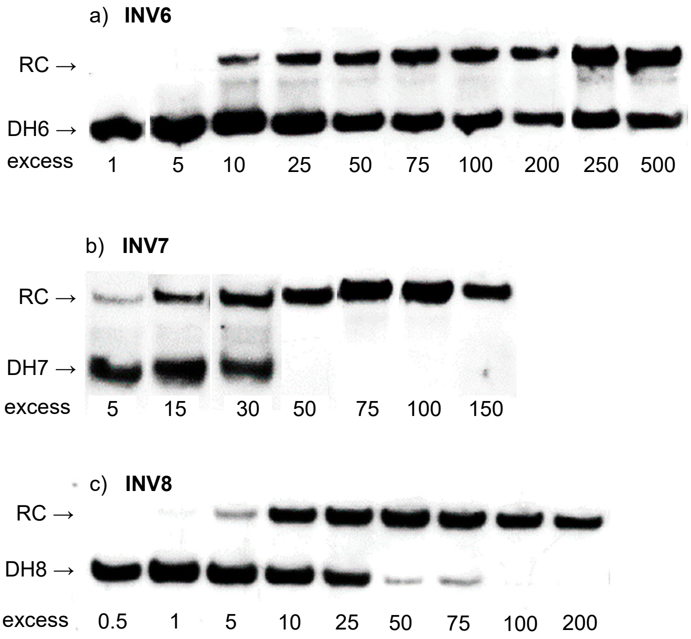

Figure S12. Dose-response experiments. Representative electrophoretograms for recognition of model DNA hairpin targets (34.4 nM) using different concentrations of the corresponding Invader probes a) **INV6**, b) **INV7**, and c) **INV8** following incubation at 37 °C for 15 h. Experimental conditions are as specified in Figure 3.

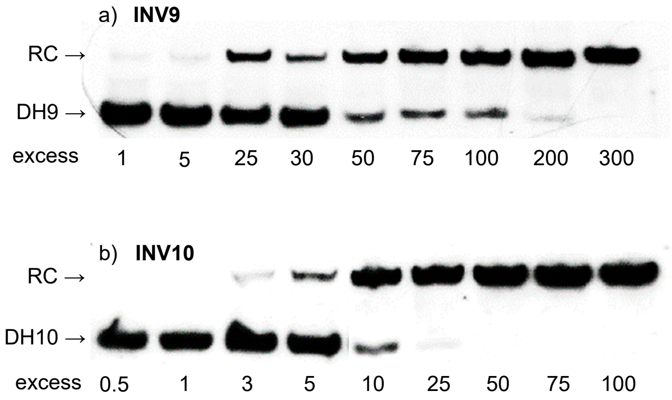

Figure S13. Representative electrophoretograms for recognition of model DNA hairpin targets (34.4 nM) using different concentrations of Invader probes a) **INV9** and b) **INV10** following incubation at 37 °C for 15 h. Experimental conditions are as specified in Figure 3.

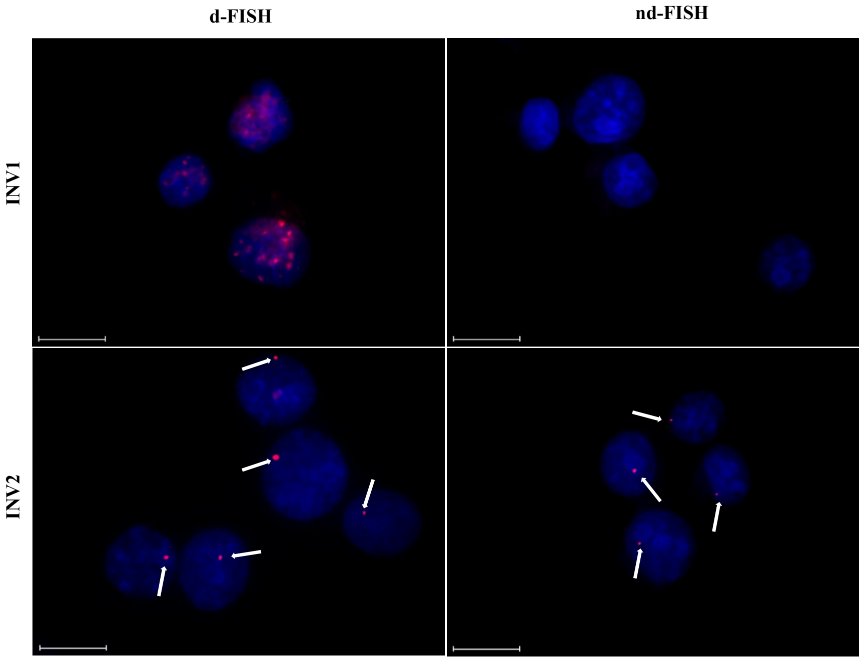

Figure S14. Representative images from FISH experiments using Invader probes **INV1** and **INV2** under denaturing (5 min, 80 °C) (left) or non-denaturing (3 h, 37.5 °C) conditions (right). Images are representative of the signal intensity and background, and the size and morphology of all analyzed nuclei (~200 nuclei per probe). Fixed isolated nuclei from male bovine kidney cells were incubated with probes in a Tris buffer (20 mM Tris-Cl, 100 mM KCl, pH 8.0) and counterstained with DAPI. Images are obtained by overlaying Cy3 (red) and DAPI (blue) filter settings and adjusting the exposure. Nuclei were viewed at 60X magnification using a Nikon Eclipse T*i*-S inverted microscope. The scale bar represents 16 µm.

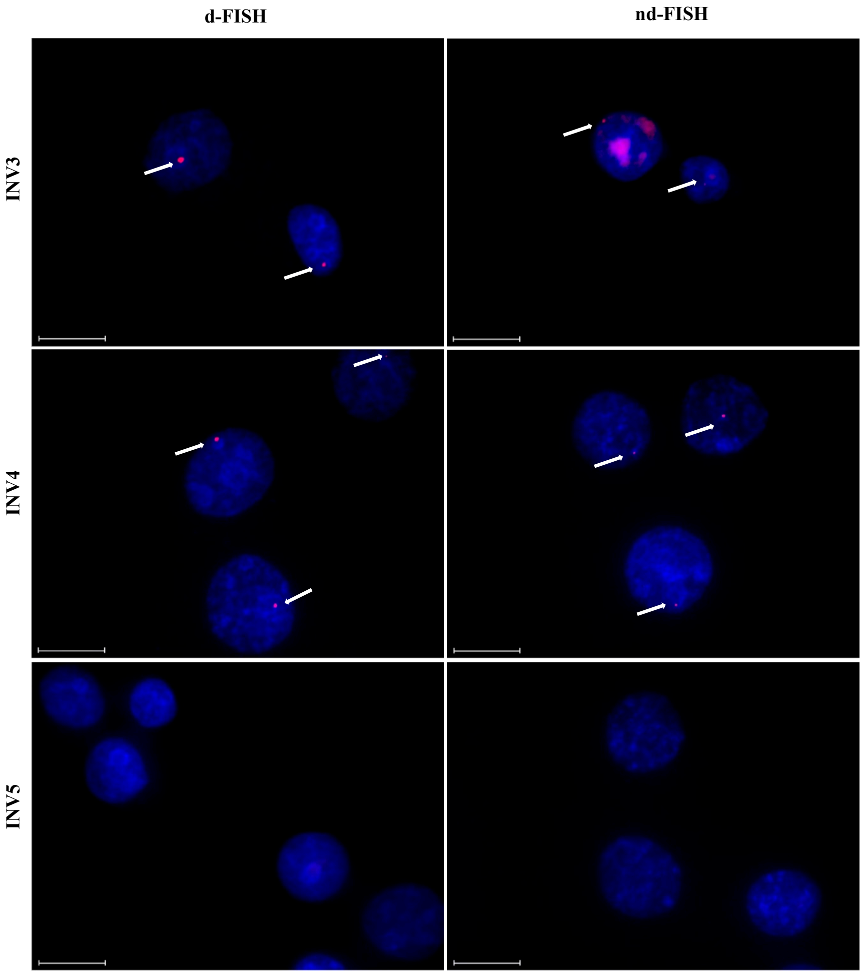

Figure S15. Representative images from FISH experiments using Invader probes **INV3**-**INV5**. Incubation conditions and the image capture process are specified in Figure S14.

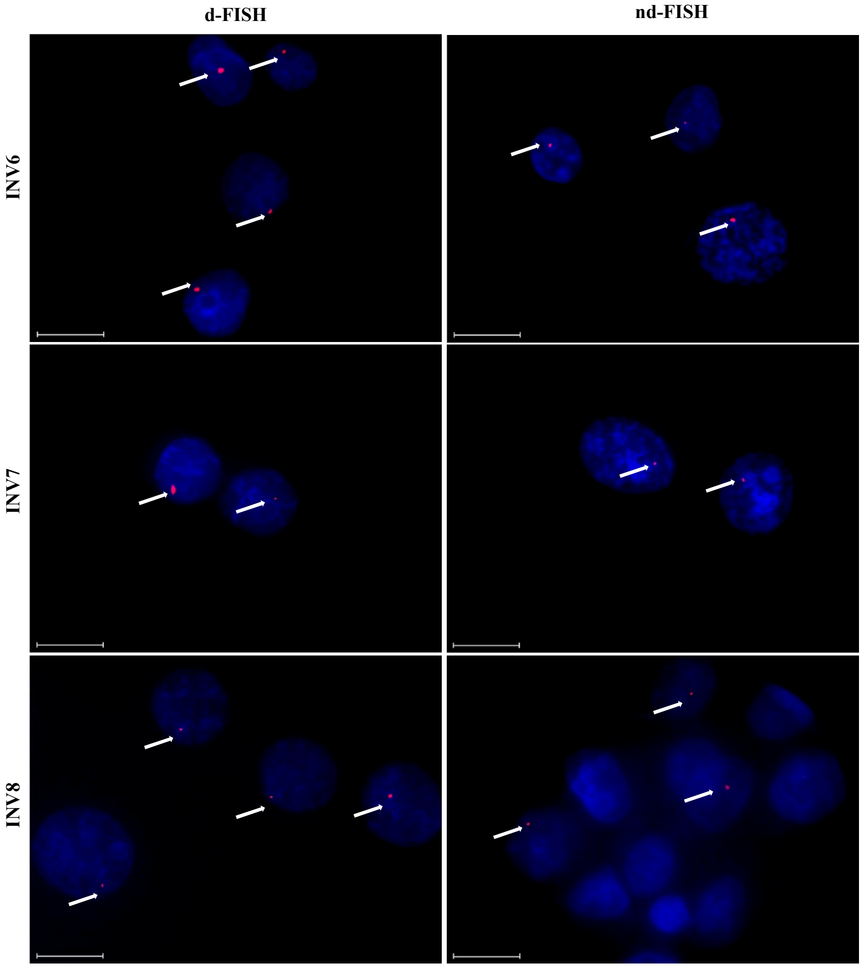

Figure S16. Representative images from FISH experiments using Invader probes **INV6**-**INV8**. Incubation conditions and the image capture process are specified in Figure S14.

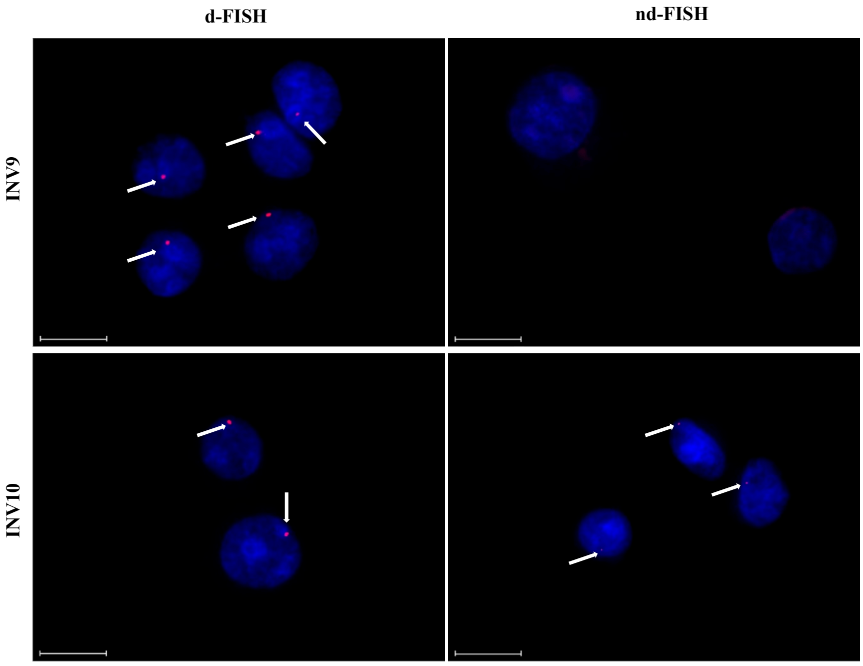

Figure S17. Representative images from FISH experiments using Invader probes **INV9** and **INV10**. Incubation conditions and the image capture process are specified in Figure S14.

INV2 INV10

DNase I

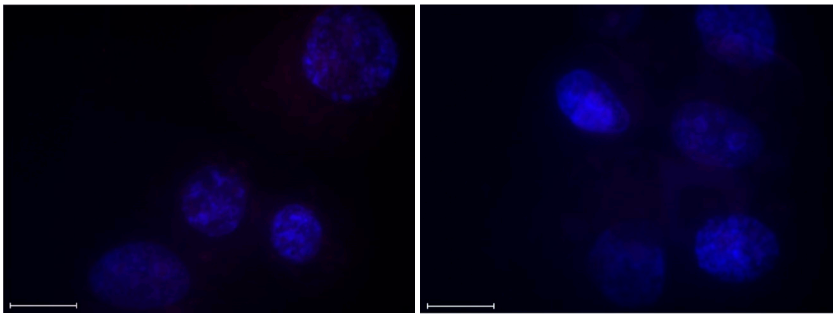

Figure S18. Representative images from nd-FISH experiments of nuclei pre-treated with DNase I prior to incubation with **INV2** or **INV10**. Note the absence of signal, which indicates that the Invader probes target chromosomal DNA. Incubation conditions and the image capture process are specified in Figure S14.

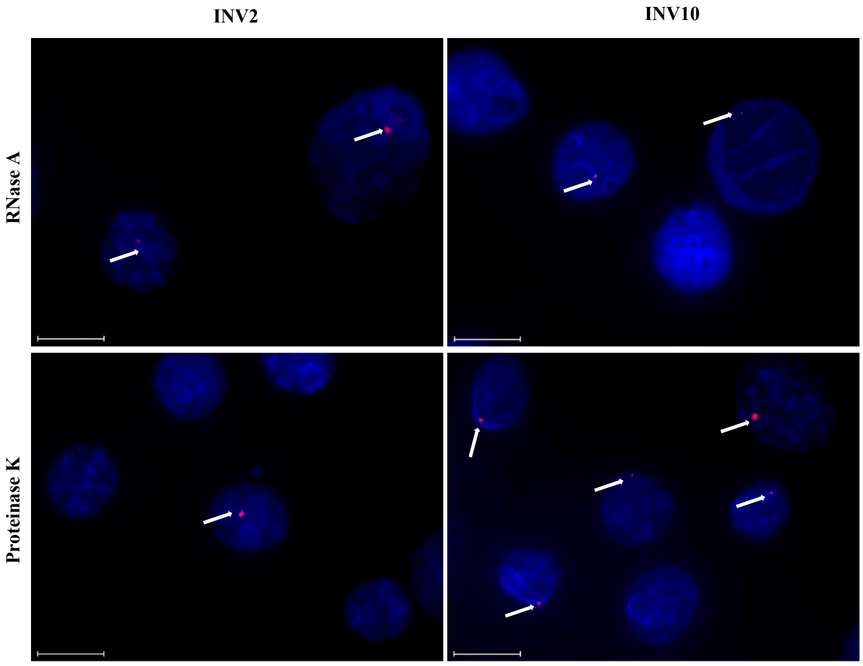

Figure S19. Representative images from nd-FISH experiments of nuclei pre-treated with RNase A or Proteinase K prior to incubation with **INV2** or **INV10**. Note the continued presence of signal in the pre-treated nuclei, which indicates that RNA or proteins are not the target of Invader probes (there is a slight reduction in signal coverage, which likely is attributed to loss of genetic material/number of nuclei due to the enzymatic treatment). Incubation conditions and the image capture process are specified in Figure S14.

Table S8. MALDI-MS of individual optimized Invader probe strands.[a]

ON	Sequence	Obs. *m/z* $[M+H]^+$	Calc. *m/z* $[M+H]^+$
**OPT6u**	5'-Cy3-C**U**G**U**G**C**AAC**U**GGT**U**TG-3'	6506	6504
**OPT6d**	3'- GA**C**A**C**G**U**TGA**C**CAA**A**C-Cy3-5'	6504	6502
**OPT8u**	5'-Cy3-TT**C**A**C**AGCCC**U**G**U**GC-3'	5905	5903
**OPT8d**	3'- AAG**U**G**U**CGGGA**C**A**C**G-Cy3-5'	6042	6041
**OPT9u**	5'-Cy3-T**U**A**U**A**U**GC**U**G**U**TCTC-3'	6114	6112
**OPT9d**	3'- AA**U**A**U**A**C**GA**C**A**A**GAG-Cy3-5'	6248	6247

[a] Individual strands are denoted up (u) or down (d).

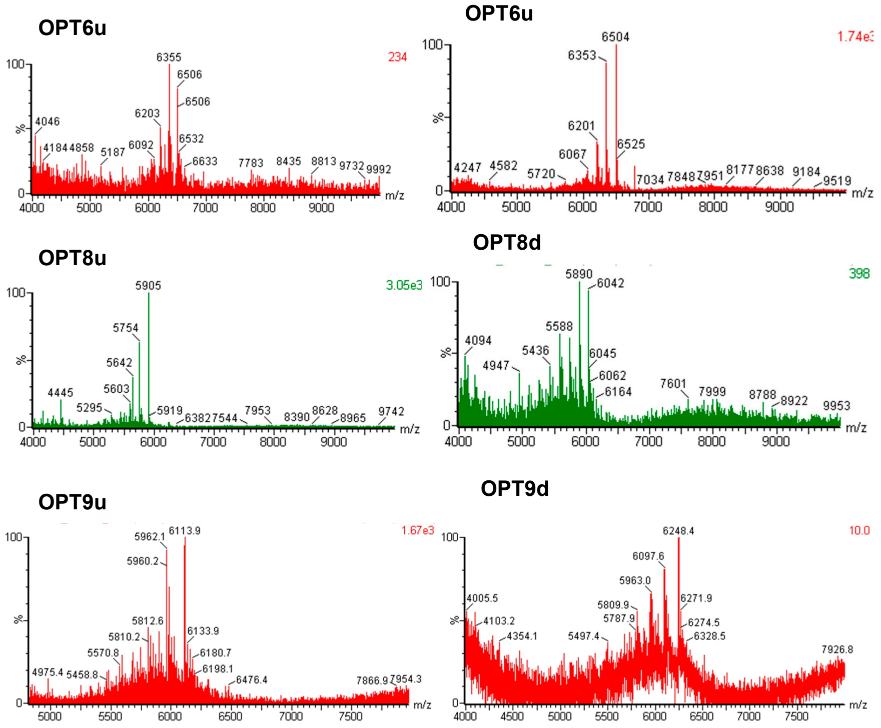

Figure S20. MALDI-MS spectra of individual Invader probe strands **OPT6**, **OPT8**, and **OPT9**.

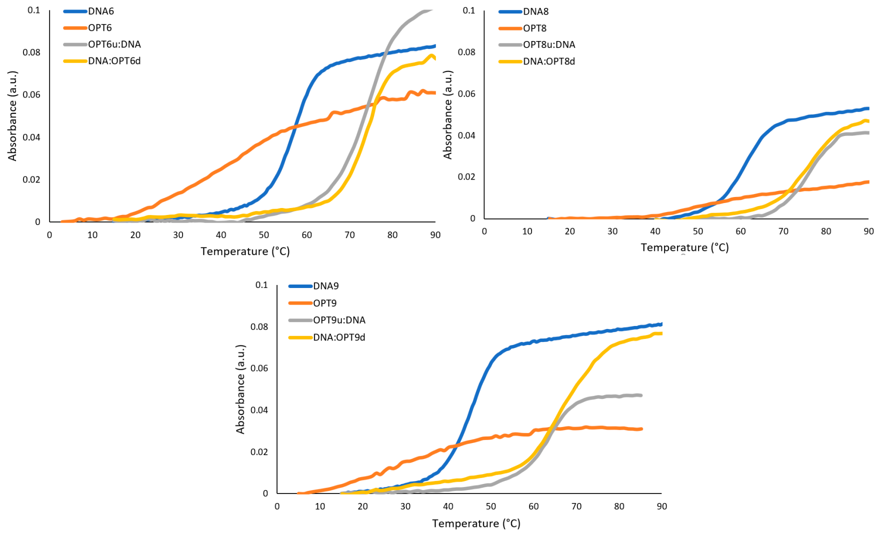

Figure S21. Representative thermal denaturation curves for optimized Invader probes **OPT6**, **OPT8**, and **OPT9**, the corresponding duplexes between individual probe strands and cDNA, and unmodified reference DNA duplexes (**DNA6**, **DNA8**, and **DNA9**).

Table S9. Change in Gibbs free energy (ΔG^{310}) upon formation of optimized Invader probes and duplexes between individual probe strands and cDNA. Also shown is the calculated change in reaction free energy upon Invader-mediated recognition of isosequential dsDNA targets (ΔG^{310}_{rec}).[a]

**Probe**	**Sequence**	**ΔG^{310}[$\Delta\Delta G^{310}$] (kJ/mol)**			**ΔG^{310}_{rec} (kJ/mol)**
		**Probe duplex**	**5'ON: cDNA**	**3'ON: cDNA**	
**OPT6**	5'-Cy3-C**U**G**U**G**C**AAC**U**GGT**U**TG-3'<br>3'- GA**C**A**C**G**U**TGA**C**CAA**A**C-Cy3-5'	-44<br>[+28]	-95<br>[-23]	-105<br>[-33]	-84
**OPT8**	5'-Cy3-TT**C**A**C**AGCCC**U**G**U**GC-3'<br>3'- AAG**U**G**U**CGGGA**C**A**C**G-Cy3-5'	-41<br>[+25]	-101<br>[-31]	-104<br>[-33]	-93
**OPT9**	5'-Cy3-T**U**A**U**A**U**GC**U**G**U**TCTC-3'<br>3'- AA**U**A**U**A**C**GA**C**A**A**GAG-Cy3-5'	-38<br>[+14]	-74<br>[-22]	-75<br>[-23]	-59

[a]$\Delta\Delta G^{310}$ is measured relative to the corresponding unmodified DNA duplex (**DNA6** = -72 kJ/mol, **DNA8** = -71 kJ/mol, **DNA9** = -52 kJ/mol).

Table S10. Change in enthalpy (ΔH) upon formation of optimized Invader probes and duplexes between individual probe strands and cDNA. Also shown is the calculated change in reaction enthalpy upon Invader-mediated recognition of isosequential dsDNA targets (ΔH_{rec}).[a]

Probe	Sequence	ΔH[ΔΔH] (kJ/mol) Probe duplex	5'ON: cDNA	3'ON: cDNA	ΔH_{rec} (kJ/mol)
**OPT6**	5'-Cy3-C**U**G**U**G**C**AAC**U**GGT**U**TG-3'<br>3'- GA**C**A**C**G**U**TGA**C**CAA**A**C-Cy3-5'	-193<br>[+327]	-519<br>[+1]	-620<br>[-100]	-426
**OPT8**	5'-Cy3-TT**C**A**C**AGCCC**U**G**U**GC-3'<br>3'- AAG**U**G**U**CGGGA**C**A**C**G-Cy3-5'	-191<br>[+280]	-555<br>[-84]	-562<br>[-91]	-455
**OPT9**	5'-Cy3-T**U**A**U**A**U**GC**U**G**U**TCTC-3'<br>3'- AA**U**A**U**A**C**GA**C**A**A**GAG-Cy3-5'	-166<br>[+297]	-476<br>[-13]	-425<br>[+38]	-272

[a]ΔΔH is measured relative to the corresponding unmodified DNA duplex (**DNA6** = -520 kJ/mol, **DNA8** = -471 kJ/mol, **DNA9** = -463 kJ/mol).

Table S11. Change in entropy (-$T\Delta S^{310}$) upon formation of optimized Invader probes and duplexes between individual probe strands and cDNA. Also shown is the calculated change in reaction entropy upon Invader-mediated recognition of isosequential dsDNA targets (-$T\Delta S^{310}_{rec}$).[a]

		**-$T\Delta S^{310}$ [$\Delta(T\Delta S^{310})$] (kJ/mol)**			
**Probe**	**Sequence**	**Probe duplex**	**5'ON: cDNA**	**3'ON: cDNA**	**-$T\Delta S^{310}_{rec}$ (kJ/mol)**
**OPT6**	5'-Cy3-CUGUGCAACUGGTUTG-3'<br>3'- GACACGUTGACCAAAC-Cy3-5'	149<br>[-299]	424<br>[-24]	515<br>[+67]	342
**OPT8**	5'-Cy3-TTCACAGCCCUGUGC-3'<br>3'- AAGUGUCGGGACACG-Cy3-5'	150<br>[-250]	452<br>[+52]	458<br>[+58]	360
**OPT9**	5'-Cy3-TUAUAUGCUGUTCTC-3'<br>3'- AAUAUACGACAAGAG-Cy3-5'	147<br>[-264]	402<br>[-9]	349<br>[-62]	193

[a]$\Delta(T\Delta S^{310})$ is measured relative to the corresponding unmodified DNA duplex (**DNA6** = 448 kJ/mol, **DNA8** = 400 kJ/mol, **DNA9** = 411 kJ/mol).

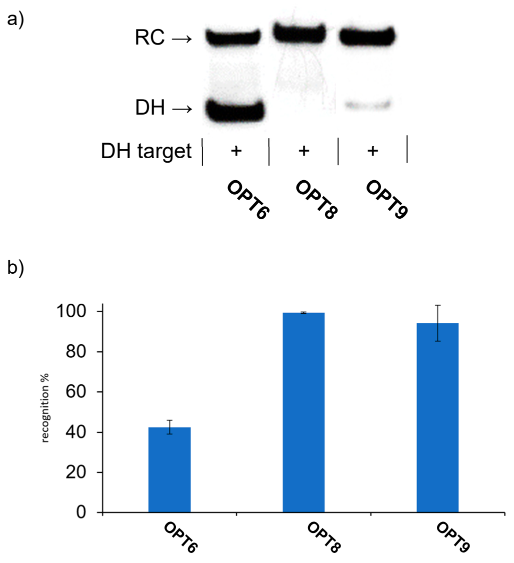

Figure S22. a) Representative gel electrophoretograms from recognition experiments in which a 100-fold molar excess of optimized Invader probes **OPT6**, **OPT8**, and **OPT9** was incubated with their respective DNA hairpin targets **DH6**, **DH8**, and **DH9** for 15h at 37 °C. b) Histograms depict averaged results from at least three recognition experiments with error bars denoting standard deviation. RC = recognition complex. DH – DNA hairpin. Incubation conditions are as described in Figure 3.

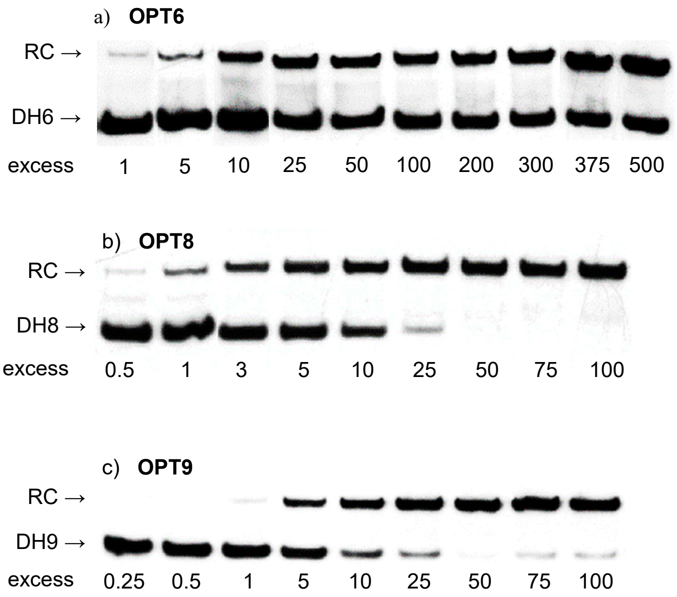

Figure S23. Dose-response experiments. Representative electrophoretograms for recognition of model DNA hairpin targets (34.4 nM) using different concentrations of the corresponding optimized Invader probes a) **OPT6**, b) **OPT8**, c) **OPT9** following incubation at 37 °C for 15 h. Experimental conditions are as specified in Figure S7. For corresponding dose-response curves, see Figure S24.

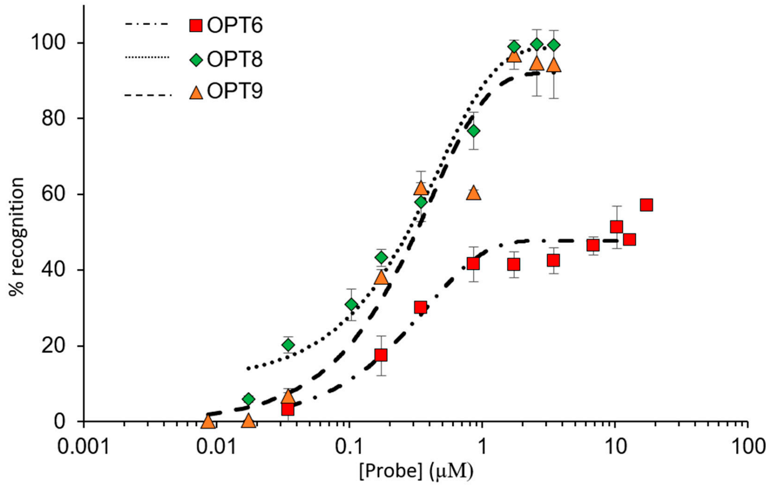

Figure S24. Dose-response curves for **OPT6**, **OPT8**, and **OPT9** following incubation with their corresponding DNA hairpin targets **DH6**, **DH8**, and **DH9** at 37 °C for 15 h. Experimental conditions are as described in Figure S7, except for variable probe concentrations. For corresponding gel electrophoretograms, see Figure S23.

Supplementary references.

[S1] Perret, J.; Shia, Y.C; Fries, R.; Vassart, G.; Georges, M. A polymorphic satellite sequence maps to the pericentric region of the bovine Y-chromosome. *Genomics,* **1990**, *6*, 482-490.

[S2] Emehiser, R.G.; Dhuri, K.; Shepard, C.; Karmakar, S.; Bahal, R.; Hrdlicka, P.J., Serine-γPNA, Invader probes, and chimeras thereof: Three probe chemistries that enable sequence-unrestricted recognition of double-stranded DNA. *Org*. *Biomol*. *Chem*., DOI: 10.1039/D2OB01567F.

[S3] Guenther, D.C.; Anderson, G.H.; Karmakar, S.; Anderson, B.A.; Didion, B.A.; Guo, W.; Verstegen, J.P.; Hrdlicka, P.J. Invader probes: harnessing the energy of intercalation to facilitate recognition of chromosomal DNA for diagnostic applications. *Chem. Sci.,* **2015**, *6*, 5006-5015.
